# HDAC8 Enhances the Function of HIF‐2α by Deacetylating ETS1 to Decrease the Sensitivity of TKIs in ccRCC

**DOI:** 10.1002/advs.202401142

**Published:** 2024-07-29

**Authors:** Kang Qian, Wei Li, Shangqing Ren, Weilin Peng, Bei Qing, Xinlin Liu, Xiong Wei, Liang Zhu, Yapeng Wang, Xin Jin

**Affiliations:** ^1^ Department of Urology, The Second Xiangya Hospital Central South University Changsha Hunan 410011 China; ^2^ Key Laboratory of Diabetes Immunology (Central South University), Ministry of Education National Clinical Research Center for Metabolic Disease Changsha 410011 China; ^3^ Department of Neurosurgery, Union Hospital, Tongji Medical College Huazhong University of Science and Technology Wuhan 430022 China; ^4^ Robotic Minimally Invasive Surgery Center, Sichuan Provincial People's Hospital, School of Medicine University of Electronic Science and Technology of China Chengdu 610072 China; ^5^ Department of Thoracic Surgery The Second Xiangya Hospital of Central South University Changsha Hunan 410011 China; ^6^ Department of Oncology, The Second Xiangya Hospital Central South University Changsha Hunan 410011 China

**Keywords:** ccRCC, ETS1, HDAC8, TKIs

## Abstract

Drug resistance after long‐term use of Tyrosine kinase inhibitors (TKIs) has become an obstacle for prolonging the survival time of patients with clear cell renal cell carcinoma (ccRCC). Here, genome‐wide CRISPR‐based screening to reveal that HDAC8 is involved in decreasing the sensitivity of ccRCC cells to sunitinib is applied. Mechanically, HDAC8 deacetylated ETS1 at the K245 site to promote the interaction between ETS1 and HIF‐2α and enhance the transcriptional activity of the ETS1/HIF‐2α complex. However, the antitumor effect of inhibiting HDAC8 on sensitized TKI is not very satisfactory. Subsequently, inhibition of HDAC8 increased the expression of NEK1, and up‐regulated NEK1 phosphorylated ETS1 at the T241 site to promote the interaction between ETS1 and HIF‐2α by impeded acetylation at ETS1‐K245 site is showed. Moreover, TKI treatment increased the expression of HDAC8 by inhibiting STAT3 phosphorylation in ccRCC cells is also found. These 2 findings highlight a potential mechanism of acquired resistance to TKIs and HDAC8 inhibitors in ccRCC. Finally, HDAC8‐in‐PROTACs to optimize the effects of HDAC8 inhibitors through degrading HDAC8 and overcoming the resistance of ccRCC to TKIs are synthesized. Collectively, the results revealed HDAC8 as a potential therapeutic candidate for resistance to ccRCC‐targeted therapies.

## Graphical abstract

TKI treatment increases HDAC8 by inhibiting STAT3 phosphorylation in ccRCC cells. Upregulated HDAC8 deacetylates ETS1 to enhance the function of the ETS1/HIF‐2α complex and decrease the sensitivity of ccRCC cells to TKI. Inhibition of HDAC8 also increases NEK1 to phosphorylate ETS1 and impede ETS1 acetylation mediated by HDAC8, so inhibiting HDAC8 on sensitized TKI is not very satisfactory.

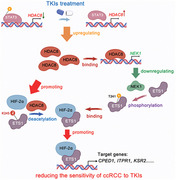

## 1 Introduction

Clear cell renal cell carcinoma (ccRCC) is the main subtype of renal cell carcinoma (RCC), accounting for approximately 85% of all RCC cases.^[^ ^1^, ^2^ ^]^ Studies have shown that most ccRCC patients have mutations in and functional inactivation of the Von Hippel Lindau (VHL) gene.^[^ ^3^ ^]^ Early inactivation of VHL leads to increased expression of intracellular hypoxia‐inducible factor alpha (HIFα) and vascular endothelial growth factor (VEGF), which further promotes ccRCC angiogenesis and tumor progression.^[^ ^4^, ^5^ ^]^ Tyrosine kinase inhibitors (TKIs), such as sunitinib and lenvatinib, inhibit tumor angiogenesis and tumor growth and have been recognized as primary agents for advanced ccRCC.^[^ ^6^ ^]^ Primary resistance or acquired resistance after long‐term use of TKIs has become an obstacle for prolonging the survival time of patients with ccRCC.^[^ ^7^ ^]^ Understanding the underlying mechanisms that regulate the sensitivity of ccRCC cells to TKIs would reveal strategies for increasing TKI sensitivity and ultimately prolonging survival in patients with ccRCC.

Protein posttranslational modifications (PTMs), such as phosphorylation, methylation, S‐palmitoylation, and acetylation, regulate cellular activity through covalent addition of functional groups or proteins, proteolytic cleavage of regulatory subunits, or degradation of proteins. These different PTMs are widely involved in regulating biological activity and further increasing the functional diversity of the proteome.^[^ ^8^, ^9^ ^]^ In recent years, an increasing number of PTMs have been found to play important roles in tumor resistance.^[^ ^10^, ^11^ ^]^ Acetylation is a widely occurring PTM, the main mechanism of which is the transfer of acetyl groups to proteins by acetyl coenzyme A (acetyl‐CoA) under the catalysis of acetyltransferase.^[^ ^12^ ^]^ Lysine acetylation is the most prominent form of acetylation and is regulated by acetyltransferase and deacetylase.^[^ ^12^ ^]^ Protein acetylation was first observed among histones more than 50 years ago, and since then, numerous cellular functions, including signal transmission, chromatin remodeling, DNA replication and repair, protein stability and transport, and transcriptional control, have been linked to this process.^[^ ^13^ ^]^ It has been reported that histone acetylation is closely associated with drug resistance,^[^ ^14^, ^15^ ^]^ for instance, TKI resistance,^[^ ^16^ ^]^ in cancer. Subsequently, lysine residues of nonhistone proteins have also been discovered and have received increased attention for their extensive regulatory functions.^[^ ^17^, ^18^ Importantly, acetylation at nonhistone proteins is critical for modulating antitumor drug sensitivity in cancers.^[^ ^19^, ^20^, ^21^ ^]^

Histone deacetylases (HDACs) constitute a family of 18 genes that are grouped into classes I–IV based on their homology to their respective yeast orthologs.^[^ ^22^ ^]^ Classes I, II, and IV consist of 11 family members that are referred to as “classical” HDACs, whereas the 7 class III members are called sirtuins.^[^ ^22^ ^]^ HDACs are involved in the regulation of the biological behaviors of cancer cells, such as the cell cycle, differentiation, apoptosis, migration, invasion, and angiogenesis, and the expression of specific HDAC family members is associated with cancer progression and patient survival in multiple cancer types.^[^ ^23^ ^]^ HDAC inhibitors are effective drugs that can induce acetylation of histones on lysine residues and inhibit tumor progression by regulating cell cycle arrest, chemotherapy sensitization, induction of apoptosis, inhibition of angiogenesis, and upregulation of tumor suppressor factors.^[^ ^24^ ^]^ Previous studies have shown that nonselective HDACis can effectively inhibit the metabolic activity of RCC cells and enhance sensitivity to sunitinib.^[^ ^25^, ^26^ ^]^ A phase I clinical study revealed that inhibiting histone deacetylase could reverse TKI resistance.^[^ ^27^ ^]^ However, nonselective HDACis may cause many side effects, such as myelosuppression and coagulation dysfunction.^[^ ^28^ ^]^ Therefore, further clarifying which HDAC family members are specifically involved in regulating the sensitivity of TKI can not only provide drug targets for ccRCC, but also reduce drug side effects for patients.

In this study, we applied genome‐wide CRISPR‐based screening to find histone deacetylase 8 (HDAC8) is involved in modulating the sensitivity of ccRCC cells to sunitinib. HDAC8 is a unique Class I HDAC that recognizes both histone and nonhistone substrates.^[^ ^29^ ^]^ Overexpression of HDAC8 has been observed in a variety of cancers (colon cancer, lung cancer, breast cancer, pancreatic cancer, optic neuroblastoma, etc.), and HDAC8 has become an important drug target for the treatment of a variety of diseases.^[^ ^29^ ^]^ However, whether HDAC8 and specific HDAC8 inhibitors are involved in the regulation of ccRCC sensitivity to sunitinib has not been reported. Therefore, understanding the effect of HDAC8 on resistance to ccRCC‐targeted therapies and elucidating the underlying mechanisms are critical for identifying potential therapeutic targets for ccRCC.

## 2 Results

### 2.1 HDAC8 Contributes to TKI Resistance in ccRCC

**Figure 1:**
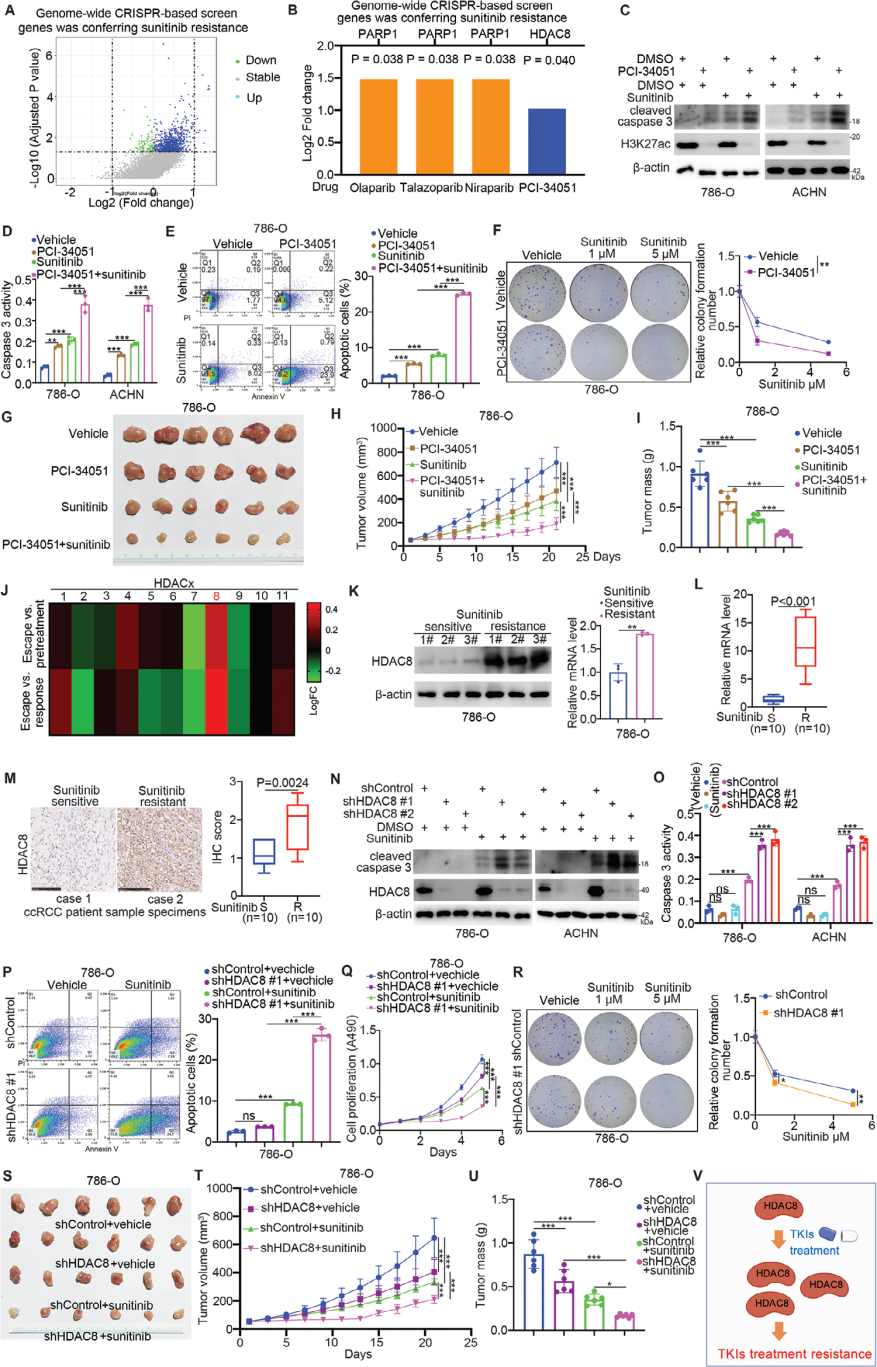
HDAC8 contributes to the resistance of ccRCC to TKIs. A and B) 786‐O cells were subjected to genome‐wide CRISPR‐Cas9 screening after lentiviral transfection and sunitinib treatment to identify genes related to drug resistance. C and D) 786‐O and ACHN cells were treated with DMSO, 10 µM PCI‐34051, or sunitinib for 24 h, and the cells were collected for western blot detection (C), and the activation of caspase3 was counted (D). Data were expressed as mean ± SD, 3 replicates. **p < 0.01; ***p < 0.001. E) 786‐O cells were treated with DMSO, PCI‐34051, or sunitinib for 24 h, and cells were collected for flow cytometry analysis. Data were expressed as mean ± SD, 3 replicates. ***p < 0.001. F) 786‐O cells were treated with DMSO, 10 µM PCI‐34051, 1 µM sunitinib, or 5 µM sunitinib for 24 h, and the cells were collected for colony formation experiments. Data were expressed as mean ± SD, 3 replicates. **p < 0.01. G–I) 786‐O cells were collected and injected subcutaneously into the back of nude mice. These mice were treated with PCI‐34051 (4 mg mL^−1^, intraperitoneal injection, every other day for 6 days) or sunitinib (80 mg Kg^−1^, oral administration, every day for 6 days). The tumor volume was measured regularly (H). The mice were sacrificed at appropriate times to remove the tumors and weigh the tumors (I). Data were expressed as mean ± SD, 6 replicates. ***p < 0.001. J) Expression levels of HDACs during sunitinib pretreatment (*n* = 4), response (*n* = 4), and resistance (escape = 4) phases. The heatmap shows the log‐fold change (LogFC) of escape versus pretreatment and the log‐fold change of escape versus response. K) 786‐O sunitinib‐sensitive and resistant cell lines were used for western blot analysis and RT‐qPCR analysis. Data were presented as mean ± SD, 3 replicates. **p < 0.01. L and M) Sunitinib‐sensitive and ‐resistant patient specimens were analyzed by RT‐qPCR (L) and immunohistochemistry (M). N and O) 786‐O and ACHN cells were transfected with indicated shRNAs for 72 h, and then treated with DMSO or 5 µM sunitinib for 24 h. The cells were collected for western blot analysis (N) and measuring the caspase 3 activity (O). For measuring the caspase 3 activity, data were expressed as mean ± SD, 3 replicates. ***p < 0.001; ns, not significant. P–R) 786‐O cells were transfected with indicated shRNAs for 72 h and then treated with DMSO or 5 µM sunitinib for 24 h. The cells were collected for flow cytometry (P), CCK8 experiment, (Q) or colony formation experiments (R). S–U) 786‐O cells were transfected with indicated shRNAs and injected subcutaneously into the back of nude mice. These mice were treated with or without sunitinib (80 mg Kg^−1^, oral administration, every day for 6 days). The tumor volume was measured regularly (T). The mice were sacrificed at appropriate times to remove the tumors and weigh the tumors (U). Data were expressed as mean ± SD, 3 or 6 replicates. ***p < 0.001; *p < 0.05. V, A model depicting that the upregulated HDAC8 after TKIs treatment is critical for modulating the sensitivity of ccRCC to TKIs.

First, genome‐wide CRISPR‐based screening was used to identify the candidate genes associated with the sensitivity of ccRCC cells to sunitinib (**Figure** **1**A). Among these candidate genes, PARP1 and HDAC8 were found to be correlated not only with the sensitivity of ccRCC cells to sunitinib but also with the sensitivity of these cells to specific inhibitors (Figure 1B). It has been well documented that PARP inhibitor treatment enhances the anticancer effect of TKIs.^[^ ^30^, ^31^ ^]^ Thus, we investigated whether HDAC8 inhibitors (such as PCI‐34051) modulate the sensitivity of ccRCC cells to TKIs. We showed that combination treatment with PCI‐34051 and sunitinib led to greater ccRCC cell apoptosis than did sunitinib treatment alone (Figure 1C–F; Figure S1A,B Supporting Information). The CCK‐8 assay showed that PCI‐34051 decreased the IC50 values of sunitinib, axitinib, and lenvatinib in ccRCC cells (Figure S1B–D, Supporting Information). Additionally, we demonstrated that PCI‐34051 treatment could reduce the IC50 value of sunitinib in sunitinib‐resistant 786‐O cells (786‐O R) as reported previously ^[^ ^32^ ^]^ (Figure S1A,B, Supporting Information). Moreover, in vivo and in vitro cell proliferation assays demonstrated that PCI‐34051 strengthened the antitumor effect of sunitinib on ccRCC cells (Figure 1G–I). In addition, clustering analysis of the GSE76068 dataset showed that HDAC8 was the most upregulated HDAC in a TKI‐resistant patient‐derived tumor xenograft (PDX) mouse model (Figure 1J). We also found that the sunitinib‐resistant ccRCC cells and patient samples had higher expression levels of HDAC8 than did the sunitinib‐sensitive cells (Figure 1K–M). Consistent with the above findings, depletion of HDAC8 promoted the anticancer effect of sunitinib (Figure 1N–U). Furthermore, downregulating HDAC8 decreased the IC50 values of sunitinib in 786‐O, ACHN, and 786‐O sunitinib resistance (786‐O R) cells (Figure S1E,F, Supporting Information). Then, we showed that repression of HDAC8 reduced the IC50 values of other TKIs, including axitinib and lenvatinib in 786‐O and ACHN cells (Figure S1G,H, Supporting Information). In contrast, overexpression of HDAC8 decreased the apoptotic effect of sunitinib and increased the IC50 values of sunitinib, axitinib, and lenvatinib in ccRCC cells (Figure S1I,M, Supporting Information). Thus, our data suggest that HDAC8 is critical for modulating the sensitivity of ccRCC to TKIs (Figure 1V).

### 2.2 HDAC8 is Closely Associated with Hypoxia‐Related Pathways and Promotes Angiogenesis in ccRCC

**Figure 2:**
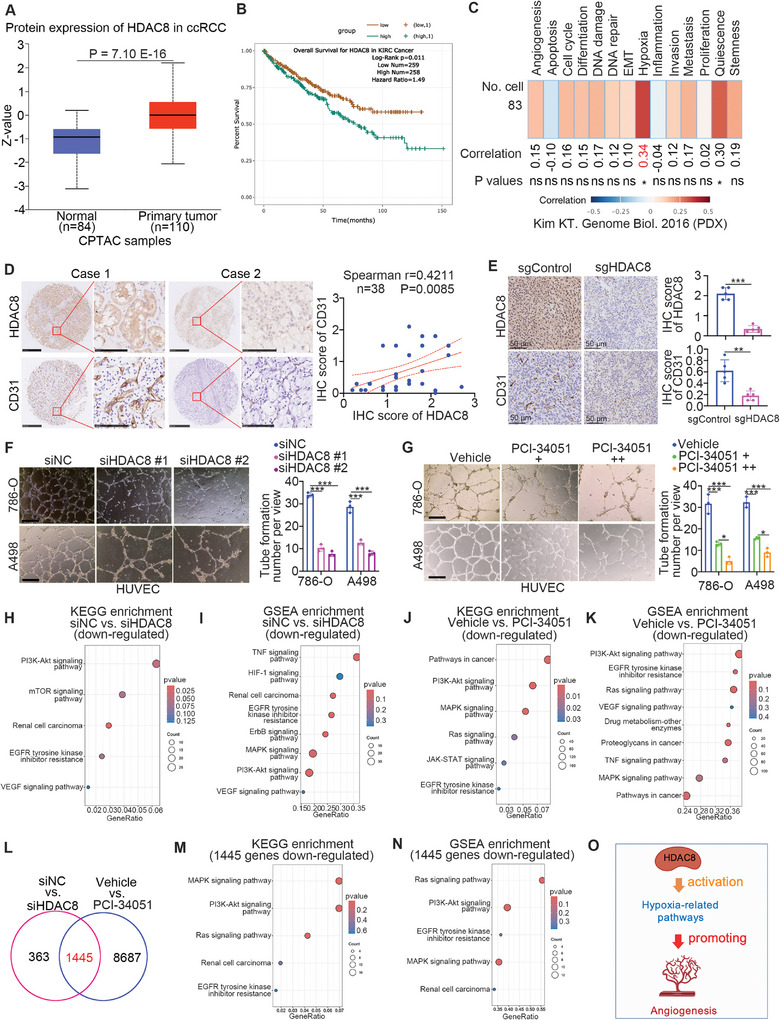
HDAC8 is closely associated with the Hypoxia‐related pathway and promotes angiogenesis of ccRCC. (A) Analyze the expression changes of HDAC8 in tumor tissues compared with normal tissues on the UALCAN website. (B) The relationship between HDAC8 expression in ccRCC tumor tissues and overall survival was analyzed using the ENCORI website. C) The correlation of HDAC8 with various phenotypes in ccRCC was analyzed using the cancerSEA website. D) The ccRCC tissue microarrays were stained with HDAC8 or CD31 antibodies. Representative images were selected for display, and the IHC score correlation between HDAC8 and CD31 was calculated. E) 786‐O cells were transfected with sgControl or sgHDAC8 for 72 h. The cells were collected for subcutaneous tumor formation on the back of nude mice. The tumor tissues were collected for immunohistochemical staining and scored for comparison. Data were expressed as mean ± SD, 5 replicates. ***p < 0.001; **p < 0.01. F) 786‐O or ACHN cells were transfected with siControl, siHDAC8#1, or siHDAC8#2 cells for 48 h, and then the supernatant and umbilical vein endothelial cells were used for angiogenesis experiments. Data were expressed as mean ± SD, 3 replicates. ***p < 0.001. G) 786‐O or ACHN cells were treated with different doses of (10 or 20 µM) PCI‐34051 for 24 h, and then the supernatant and umbilical vein endothelial cells were used for angiogenesis experiments. Data were expressed as mean ± SD with 3 replicates. ***p < 0.001; *p < 0.05. H and I) 786‐O cells were treated with siNC or siHDAC8 for 48 h. The cells were collected for transcriptome sequencing, and then KEGG enrichment analysis of down‐regulated molecules was performed (H), and GSEA enrichment analysis was performed to find down‐regulated pathways (I). J and K) 786‐O cells were treated with vehicle or 10 µM PCI‐34051 for 24 h. The cells were collected for transcriptome sequencing, and then KEGG enrichment analysis of down‐regulated molecules was performed (J), and GSEA enrichment analysis was performed to find down‐regulated pathways (K). L–O) The intersection of the changed molecules after knockdown of HDAC8 and PCI‐34051 treatment was performed (L), KEGG enrichment analysis was performed on the down‐regulated molecules among the differential molecules (M), and then GSEA analysis was performed to find the down‐regulated pathways (N). O, A model depicting that HDAC8 is responsible for activating hypoxia‐related signaling pathways and promoting angiogenesis in ccRCC.

Analysis of the CPTAC dataset revealed that the protein level of HDAC8 in renal cancer tissue was higher than that in nontumor renal tissue (**Figure** **2**A). High expression of HDAC8 was also negatively correlated with the overall survival of patients with ccRCC according to analysis of the TCGA‐KIRC dataset (Figure 2B). Dysregulation of hypoxia and angiogenesis is the major reason for the resistance of ccRCC to TKIs.^[^ ^33^ ^]^ To further investigate how HDAC8 contributes to modulating the sensitivity of ccRCC to TKIs, we applied the CancerSEA web tool (http://biocc.hrbmu.edu.cn/CancerSEA/) to study the biological function of HDAC8 in ccRCC, and this analysis showed that HDAC8 expression was positively correlated with hypoxia, stemness, metastasis, and angiogenesis but negatively correlated with apoptosis; the association of HDAC8 expression with hypoxia was significant in ccRCC according to the P value (Figure 2C; Figure S2A, Supporting Information). We also demonstrated that there was a positive correlation between HDAC8 expression and hypoxia or angiogenesis in other malignant tumors, including ovarian carcinoma, colorectal cancer, prostate cancer, and retinoblastoma, by analyzing the CancerSEA web tool (Figure S2B–E, Supporting Information). Consistent with the above findings, the IHC staining assay revealed that the protein level of HDAC8 in the ccRCC tissue microarray was positively correlated with the protein level of CD31, which is a marker of blood vessels (*n* = 38, Spearman correlation *r* = 0.4211, *P* = 0.0085) (Figure 2D). We also showed that the protein level of CD31 in the HDAC8 depletion group was lower than that in the control group after IHC staining of the xenografts (Figure 2E). Moreover, tube formation assays showed that knocking down HDAC8 or treating cells with PCI‐34051 suppressed angiogenesis in vitro (Figure 2F,G; Figure S2F, Supporting Information). Transcriptome analysis was performed after the knockdown of HDAC8 or treatment with PCI‐34051 in 786‐O cells (Figure S2G,H, Supporting Information). Kyoto Encyclopedia of Genes and Genomes (KEGG) and Gene Set Enrichment Analysis (GSEA) enrichment analysis showed that knockdown of HDAC8 inactivated the PI3K‐AKT signaling pathway, TNF signaling pathway, HIF‐1 signaling pathway, EGFR tyrosine kinase inhibitor resistance pathway, and VEGF signaling pathway (Figure 2H,I), which are important for regulating angiogenesis and TKI resistance.^[^ ^33^ ^]^ Similar pathways were inactivated after treatment with PCI‐34051, as shown in Figure 2J,K. Furthermore, 1445 genes were commonly altered between control and HDAC8 knockdown cells and between control‐ and PCI‐34051‐treated cells (Figure 2L). KEGG enrichment analysis of these 1445 genes revealed that multiple types of signaling pathways, such as those related to cellular senescence and renal cell carcinoma, were regulated by HDAC8 (Figure S2I, Supporting Information). After analyzing the downregulated genes among these 1445 genes, we showed that the PI3K‐AKT signaling pathway, EGFR tyrosine kinase inhibitor resistance pathway, and renal cell carcinoma signaling pathway were inactivated (Figure 2M,N). These results indicated that HDAC8 is responsible for promoting angiogenesis and activating multiple oncogenic signaling pathways associated with TKI resistance in ccRCC (Figure 2O).

### 2.3 HDAC8 Interacts with ETS1 to Reduce the Sensitivity of ccRCC Cells to Sunitinib

**Figure 3:**
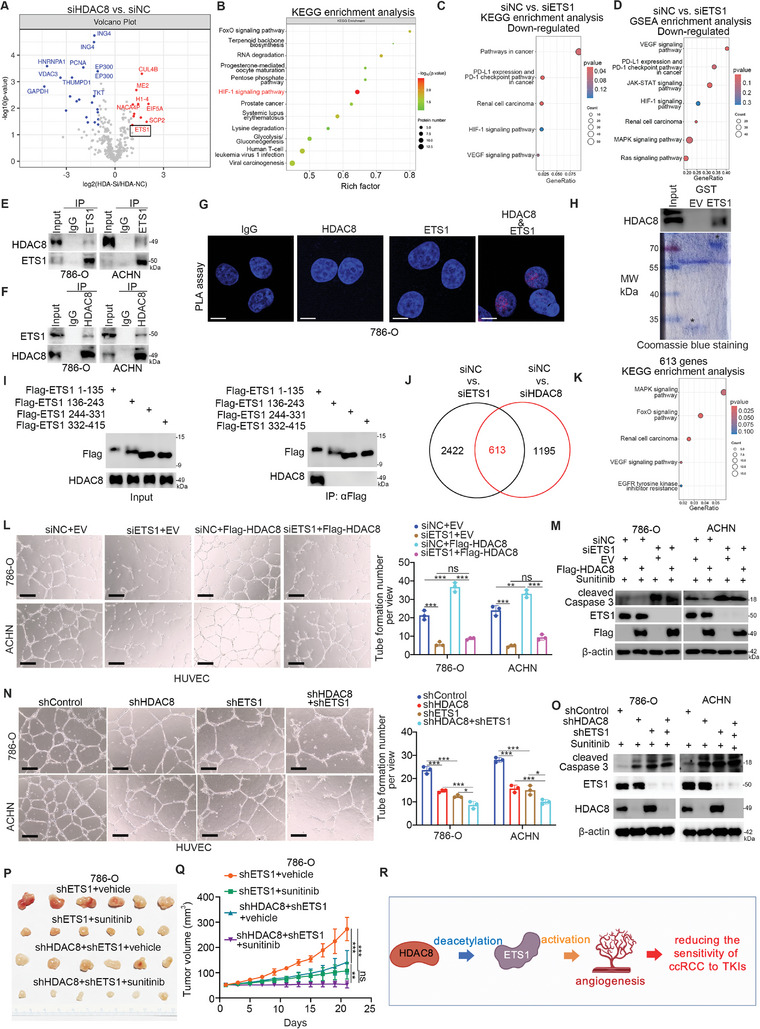
HDAC8 interacted with ETS1 to reduce the sensitivity of ccRCC cells to sunitinib. A and B) 786‐O cells were transfected with siHDAC8 or siNC for 48 h, and the cells were collected for quantitative acetylation proteomics(A), followed by KEGG analysis (B). C and D) 786‐O cells were transfected with siETS1 or siNC for 48 h, and the cells were collected for transcriptome sequencing. After finding the down‐regulated molecules, KEGG analysis was performed (C), and then GSEA analysis was performed to find the down‐regulated pathways (D). E and F) In 786‐O and ACHN cell lines, western blot detection was performed after co‐immunoprecipitation experiments with ETS1 or HDAC8 antibodies. G) Proximity ligation assay was performed in 786‐O cells using antibodies against ETS1 and HDAC8. H) Western blotting analysis of HDAC8 GST‐pulled down by ETS1 recombinant. I) A truncated form of ETS1 was constructed as indicated in the figure, and co‐immunoprecipitation was performed with Flag‐tagged antibodies, followed by western blot experiments. J and K) The molecules whose expression levels changed after knocking down HDAC8 or knocking down ETS1 were intersected (J), and KEGG enrichment analysis was performed on them (K). L and M) As indicated in the figure, 786‐O, and ACHN cells were transfected with indicated truncates. After 48 h, cell culture media were collected for angiogenesis assays (L). Cells were collected for western blot analysis after treatment with 5 µM sunitinib for 24 h (M). Data were expressed as mean ± SD with 3 replicates. ***p < 0.001; **p < 0.01; ns, not significant. N and O) As indicated in the figure, 786‐O, and ACHN cells were transfected. After 72 h, cell culture media were collected for angiogenesis assays (N). Cells were collected for western blot analysis after treatment with sunitinib for 24 h (O). Data were expressed as mean ± SD, 3 replicates. ***p < 0.001; *p < 0.05. P and Q) 786‐O cells were transfected with shETS1 for 72 h. Then, these cells were transfected with shControl or shHDAC8 for another 72 h. Cells were collected and injected subcutaneously into the back of nude mice. These mice were treated with or without sunitinib (80 mg Kg^−1^, oral administration, every day for 6 days). The tumor volume was measured regularly (Q). Data were presented as mean ± SD, 3 or 6 replicates. ***p < 0.001; **p < 0.01; ns, not significant. R) A model depicting that HDAC8 interacts with and deacetylates ETS1 to reduce the sensitivity of ccRCC cells to TKIs.

Given that HDAC8 regulates the biological functions of cells mainly through its deacetylase activity,^[^ ^29^ ^]^ we subjected 786‐O cells to proteomics after HDAC8 was knocked down to assess acetylation and identify potential nonhistone proteins that could be deacetylated by HDAC8 (**Figure** **3**A). The acetylation levels of numerous proteins, including CUL4B, SCP2, and ETS1, were increased after the inhibition of HDAC8 (Figure 3A; Table S4, Supporting Information). A CCK‐8 assay demonstrated that overexpression of ETS1 increased the IC50 of sunitinib in ccRCC cells (Figure S3A, Supporting Information). However, the overexpression of SCP2 or CUL4B had no effect on the IC50 values of sunitinib in ccRCC cells (Figure S3B,C, Supporting Information). Moreover, KEGG enrichment analysis of the differentially acetylated proteins showed that multiple types of signaling pathways were involved, especially the HIF‐1 signaling pathway (Figure 3B). It has been reported that ETS1 is upregulated by HIF‐1α and interacts with HIF‐2α to activate the HIF‐1 signaling pathway and promote angiogenesis.^[^ ^34^, ^35^ ^]^ Consistently, transcriptome analysis after ETS1 was knocked down in 786‐O cells demonstrated that the renal cell carcinoma signaling pathway, HIF‐1 signaling pathway, and VEGF signaling pathway were inactivated (Figures 3C,D, S3D). Co‐IP demonstrated that HDAC8 interacts with ETS1 in 786‐O and ACHN cells (Figure 3E,F). Immunofluorescence after proximity ligation assay (PLA) also demonstrated that HDAC8 binds to ETS1 in 786‐O cells (Figure 3G). Moreover, a GST pull‐down assay showed that HDAC8 binds to ETS1 (Figure 3H). The ETS1 can be divided into 4 components, ETS1‐1 (1‐134 aa), ETS1 (135‐242 aa), ETS1‐3 (243‐330 aa), and ETS1‐4 (331‐441 aa), according to the domain of ETS1 (Figure S3E, Supporting Information). Co‐IP analysis revealed that the ETS1‐1 (1‐134 aa) construct interacts with HDAC8 in 786‐O cells (Figure 3I). Next, we investigated whether ETS1 is the key mediator of HDAC8‐induced resistance of ccRCC cells to sunitinib. First, we found that 613 genes were commonly regulated by ETS1 and HDAC8 (Figure 3J) and were involved in the VEGF signaling pathway, and the EGFR tyrosine kinase inhibitor resistance signaling pathway (Figure 3K). Knockdown of ETS1 attenuated the effect of HDAC8 on angiogenesis and sunitinib sensitivity in ccRCC cells (Figure 3L–Q; Figure S3F–I, Supporting Information). Thus, these data suggest that ETS1 acts as a mediator of the HDAC8‐induced reduction in sunitinib sensitivity in ccRCC cells (Figure 3R).

### 2.4 HDAC8 Deacetylates ETS1 at the K245 Site in ccRCC Cells

**Figure 4:**
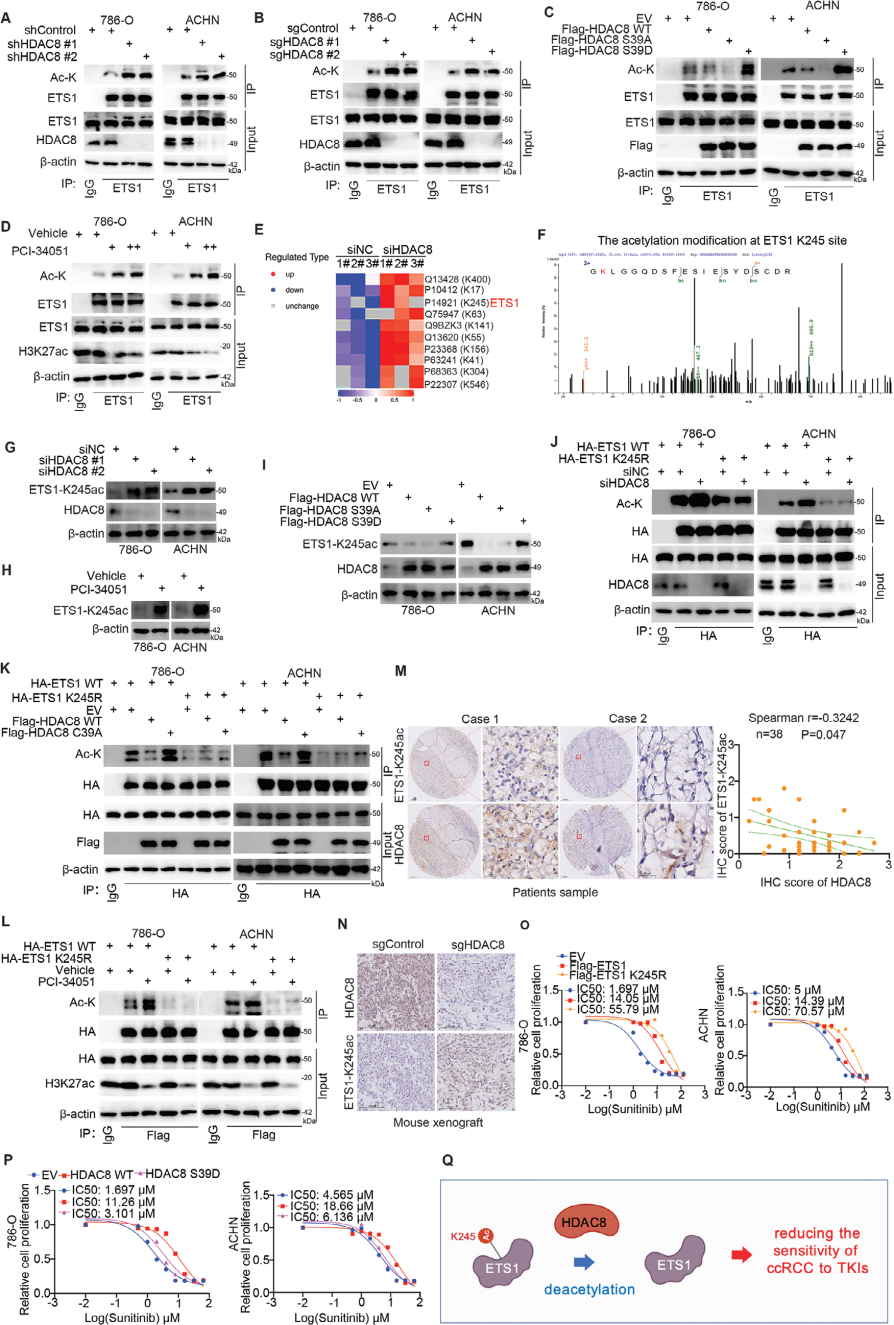
HDAC8 de‐acetylates ETS1 at the K245 site in ccRCC cells. A and B) 786‐O and ACHN cells were transfected with indicated shRNAs or sgRNAs for 72 h. Cells were collected for co‐IP and western blot analysis. C, HDAC8 mutants were constructed as indicated. After transfection as indicated for 48 h, 786‐O and ACHN cells were collected and subjected to co‐immunoprecipitation experiments with IgG or ETS1 antibodies followed by western blot analysis. D) After treating 786‐O and ACHN cells with vehicle or (+, 10 µM; ++, 20 µM) PCI‐34051 for 24 h, the cells were collected for co‐IP with IgG or ETS1 antibodies and then subjected to western blot analysis. E and F) After 786‐O was transfected with siNC or siHDAC8, quantitative acetylation proteomics was performed to identify protein modification sites (E). Peptide map of the acetylation of ETS1 at K245 site (F). G) 786‐O and ACHN cells were transfected with indicated siRNAs for 48 h, and cells were subjected to western blot analysis. H) 786‐O and ACHN cells were treated with DMSO (vehicle) or 10 µM PCI‐34051 for 24 h, and cells were subjected to western blot analysis. I) 786‐O and ACHN cells were transfected with indicated constructs for 48 h, and cells were subjected to western blot analysis. J and K) 786‐O and ACHN cells were transfected with indicated constructs for 48 h, and cells were subjected to co‐IP with IgG or HA‐tagged antibodies, and western blot analysis. L) 786‐O and ACHN cells were transfected with indicated constructs for 24 h, then cells were treated with vehicle or 10 µM PCI‐34051 for another 24 h. Cells were harvested for co‐IP and western blot analysis. M) The ccRCC tumor tissue microarray was stained with HDAC8 or ETS1‐K245ac antibodies. Representative images were selected for display, and the IHC score correlation between HDAC8 and ETS1‐K245ac was calculated. N) 786O cells were transfected with sgControl or sgHDAC8 for 72 h, and cells were subjected to xenograft assay. The tumor was stained with HDAC8 or ETS1‐K245ac antibodies. O and P) 786‐O and ACHN cells were transfected with indicated constructs for 48 h, and cells were treated with a serial dose of sunitinib for another 24 h. These cells were harvested for CCK8 assay to calculate the IC50 values. Q) A model depicting that ETS1‐K245 site is deacetylated by HDAC8 to regulate the sensitivity of ccRCC cells to TKIs.

Next, we demonstrated that depleting the expression of HDAC8 by transfecting 786‐O and ACHN cells with HDAC8‐specific shRNAs or sgRNAs increased the acetylation level of ETS1 (**Figure** **4**A,B). In contrast, overexpression of wild‐type (WT) HDAC8 or the HDAC8 S39A mutant decreased the acetylation level of ETS1, whereas overexpression of the HDAC8 deacetylate‐defective mutant (HDAC8 S39D).^[^ ^36^ ^]^ failed to reduce the acetylation level of ETS1 in ccRCC cells (Figure 4C). In addition, treatment with an HDAC8 inhibitor increased the acetylation level of ETS1 in ccRCC cells (Figure 4D). Proteomics analysis was used to assess acetylation levels after the knockdown of HDAC8 in 786‐O cells and revealed that the acetylation level of lysine 245 (K245) of ETS1 increased (Figure 4E,F). We showed that inhibiting the expression or function of HDAC8 resulted in an increase in the acetylation level of ETS1 at K245 (ETS1‐K245ac) in ccRCC cells (Figure 4G,H). In contrast, the HDAC8 WT or the HDAC8 S39A mutant but not the HDAC8 deacetylate‐defective mutant (S39D) decreased the ETS1‐K245ac level in ccRCC cells (Figure 4I). On the other hand, we also showed that altering the expression of HDAC8 or repressing the function of HDAC8 had little effect on the acetylation level of the ETS1 K245R mutant (Figure 4J–L). Furthermore, IHC staining of the ccRCC patient tissue microarray demonstrated that there was a negative correlation between HDAC8 and ETS1‐K245ac (*n* = 38, Spearman correlation *r* = −0.3242, *P* < 0.047) (Figure 4M). IHC staining also showed that the knockdown of HDAC8 increased ETS1‐K245ac levels (Figure 4N). The CCK‐8 assay indicated that the increase in the IC50 value of sunitinib after overexpression of the ETS1‐K245R mutant was greater than that after overexpression of ETS1 WT in ccRCC cells (Figure 4O). Additionally, we showed that compared to overexpression of HDAC8 WT in ccRCC cells, overexpression of the HDAC8 S39D mutant did not further increase the IC50 value of sunitinib (Figure 4P). Therefore, our results suggest that the ETS1‐K245 site is deacetylated by HDAC8, and this process is involved in regulating the sensitivity of ccRCC cells to sunitinib (Figure 4Q).

### 2.5 HDAC8‐Mediated Deacetylation of ETS1‐K245 Promotes ETS1 Binding with HIF‐2α to Enhance the Transcriptional Activity of the ETS1/HIF2A Complex in ccRCC cells

**Figure 5:**
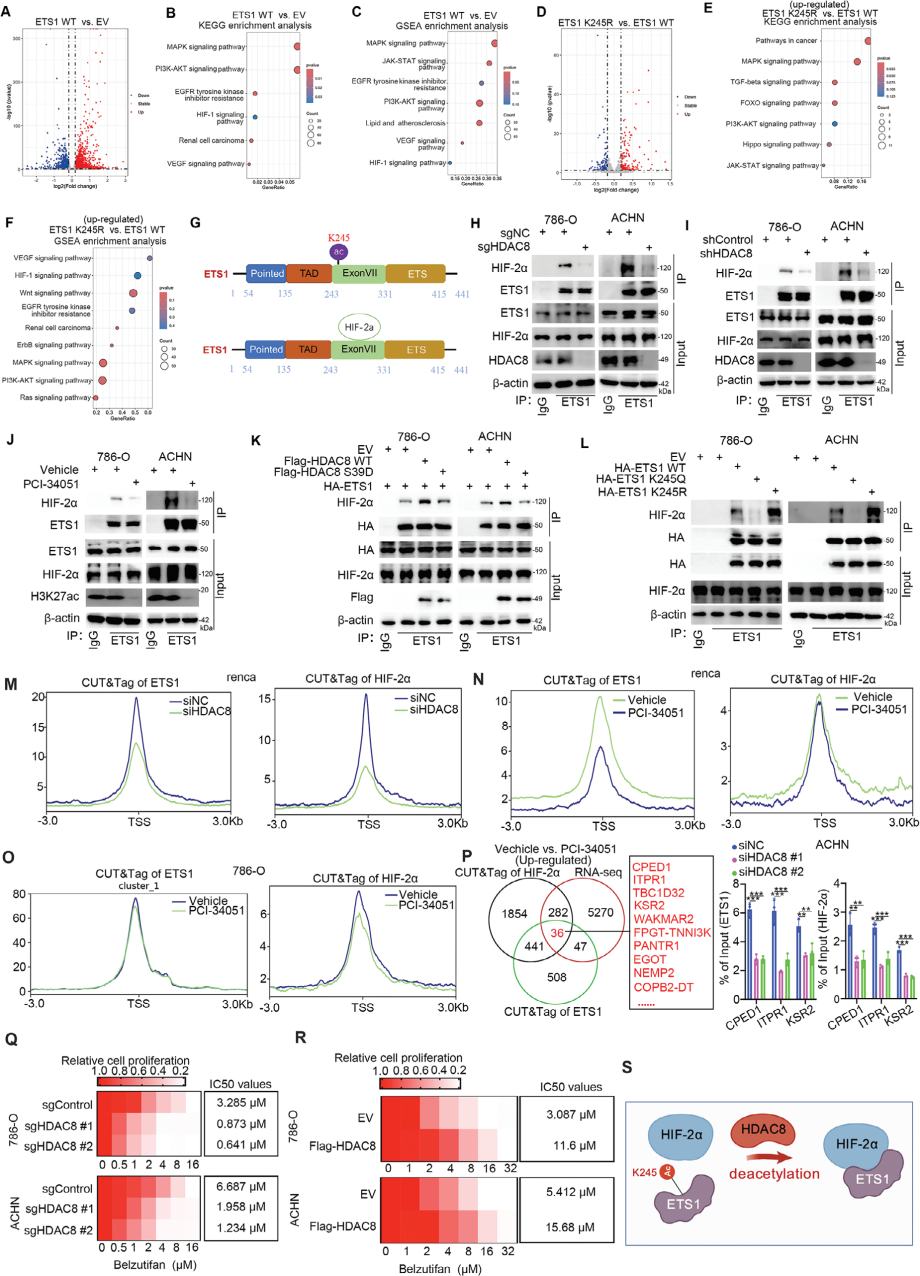
The de‐acetylation of ETS1‐K245 mediated by HDAC8 promotes ETS1 binds with HIF‐2α to enhance the transcriptional activity of ETS1/ HIF‐2α complex in ccRCC cells. A‐C, ETS1 WT, or empty vector (EV) were transfected into 786‐O cells for 48 h, and the cells were collected for transcriptome sequencing. After obtaining the differential genes A), KEGG enrichment analysis B) was performed, followed by GSEA enrichment analysis C). D–F) ETS1 K245R or ETS1 WT were transfected into 786‐O cells for 48 h. The cells were collected for transcriptome sequencing. After obtaining the differential genes (D), KEGG enrichment analysis (E) was performed, followed by GSEA enrichment analysis (F). G) A model depicting that the acetylation site of ETS (K254) was located at the ExonVII domain of ETS1, and this domain is also bound with HIF‐2a. H and I) 786‐O and ACHN cells were transfected with indicated sgRNAs or shRNAs for 72 h, and cells were harvested for co‐IP and western blot analysis. J) 786‐O and ACHN cells were treated with vehicle or 10 µM PCI‐34051 for 24 h, and cells were collected co‐IP and western blot analysis. K and L) 786‐O and ACHN cells were transfected with indicated plasmids for 48 h, and cells were harvested for co‐IP and western blot analysis. M) Renca cells were transfected with siNC or siHDAC8 for 48 h, and cells were collected for CUT&Tag by using the ETS1 or HIF‐2α antibodies. N) Renca cells were treated with vehicle or 10 µM PCI‐34051 for 24 h, and cells were collected for CUT&Tag by using the ETS1 or HIF‐2α antibodies. O) 786‐O cells were treated with vehicle or 10 µM PCI‐34051 for 24 h, and cells were collected for CUT&Tag by using the ETS1 or HIF‐2α antibodies. P) Analyzing the RNA‐seq and CUT&Tag data after being treated with PCI‐34051 in ccRCC cells. ACHN cells were transfected with indicated siRNAs for 48 h, cells were collected for ChIP‐qPCR analysis. Data were presented as mean ± SD with 3 replicates. ***p < 0.001; **p < 0.01. Q and R) 786‐O and ACHN cells were transfected with indicated sgRNAs for 72 h (Q), or indicated plasmids for 48 h (R). Cells were treated with Belzutifan at different concentration gradients for 24 h, and CCK8 experiments were performed to measure the IC50 values of Belzutifan. S) A model depicting that ETS1‐K245 site is deacetylated by HDAC8 to promote the binding of ETS1 and HIF‐2α.

We subsequently investigated how HDAC8‐mediated deacetylation of ETS1‐K245 modulates the sensitivity of ccRCC to TKIs. Consistent with the above findings, transcriptome analysis after overexpression of wild‐type ETS1 (ETS1 WT) showed that ETS1 was closely associated with the activation of the MAPK, PI3K‐AKT, EGFR TKI resistance, VEGF, and HIF‐1 signaling pathways in ccRCC cells (**Figure** **5**A–C). KEGG and GSEA enrichment analysis of the RNA‐seq data after overexpression of the ETS1‐K245R mutant, which mimics deacetylation at the K245 site, indicated that, compared with WT ETS1, ETS1‐K245R further activated the MAPK, PI3K‐AKT, EGFR TKI resistance, VEGF, and HIF‐1 signaling pathways in ccRCC cells (Figure 5D–F). The ETS1‐K245 site is located in the exon VII domain of ETS1 and is also responsible for binding with HIF‐2α.^[^ ^37^ ^]^(Figure 5G). Subsequent co‐IP assays demonstrated that depleting HDAC8 or treating ccRCC cells with PCI‐34051 decreased the binding of HIF‐2α to ETS1 (Figure 5H–J). However, overexpression of HDAC8 WT but not the functionally dead mutant (HDAC8 S39D) promoted ETS1 binding with HIF‐2α in ccRCC cells (Figure 5K). We demonstrated that the ETS1‐K245R mutant interacted more readily with HIF‐2α than did the ETS1‐WT or ETS1‐K245Q mutant, mimicking the acetylation of ETS1 (Figure 5L). It has been reported that ETS1 forms a complex with HIF‐2α to modulate downstream gene expression and activate pathways associated with angiogenesis.^[^ ^38^ ^]^ We performed CUT&Tag assays in Renca cells and 786‐O cells and found that knocking down HDAC8 or treating cells with PCI‐34051 attenuated the enrichment of ETS1 or HIF‐2α on target genes (Figure 5M–O; Figure S4A–D, Supporting Information). By combining the RNA‐seq data and CUT&Tag data after being treated with PCI‐34051 in 786‐O cells, there might be 36 genes (including CPED1, ITPR1, or KSR2) up‐regulated by the HDAC8/ETS1/HIF‐2α axis, including the known downstream gene of HIF‐2α—ITPR1^[^ ^39^ ^]^ (Figure 5P).

The subsequent ChIP‐qPCR assay showed that the knockdown of HDAC8 decreased the binding of ETS1 or HIF‐2α on these genes’ promoters, such as CPED1, ITPR1, or KSR2 (Figure 5P; Figure S4E, Supporting Information). Furthermore, we showed that changing the expression of HDAC8 or treated PCI‐34051 regulated the anticancer effect of a HIF‐2α inhibitor (PT2385) on ccRCC cells (Figure 5Q,R; Figure S4F, Supporting Information). Thus, these data suggest that HDAC8 deacetylates ETS1‐K245 to strengthen the ability of the ETS1/HIF‐2α complex to modulate angiogenesis (Figure 5S).

### 2.6 NEK1, Repressed by HDAC8, Phosphorylates ETS1 to Enhance the Binding Between ETS1 and HIF‐2α

**Figure 6:**
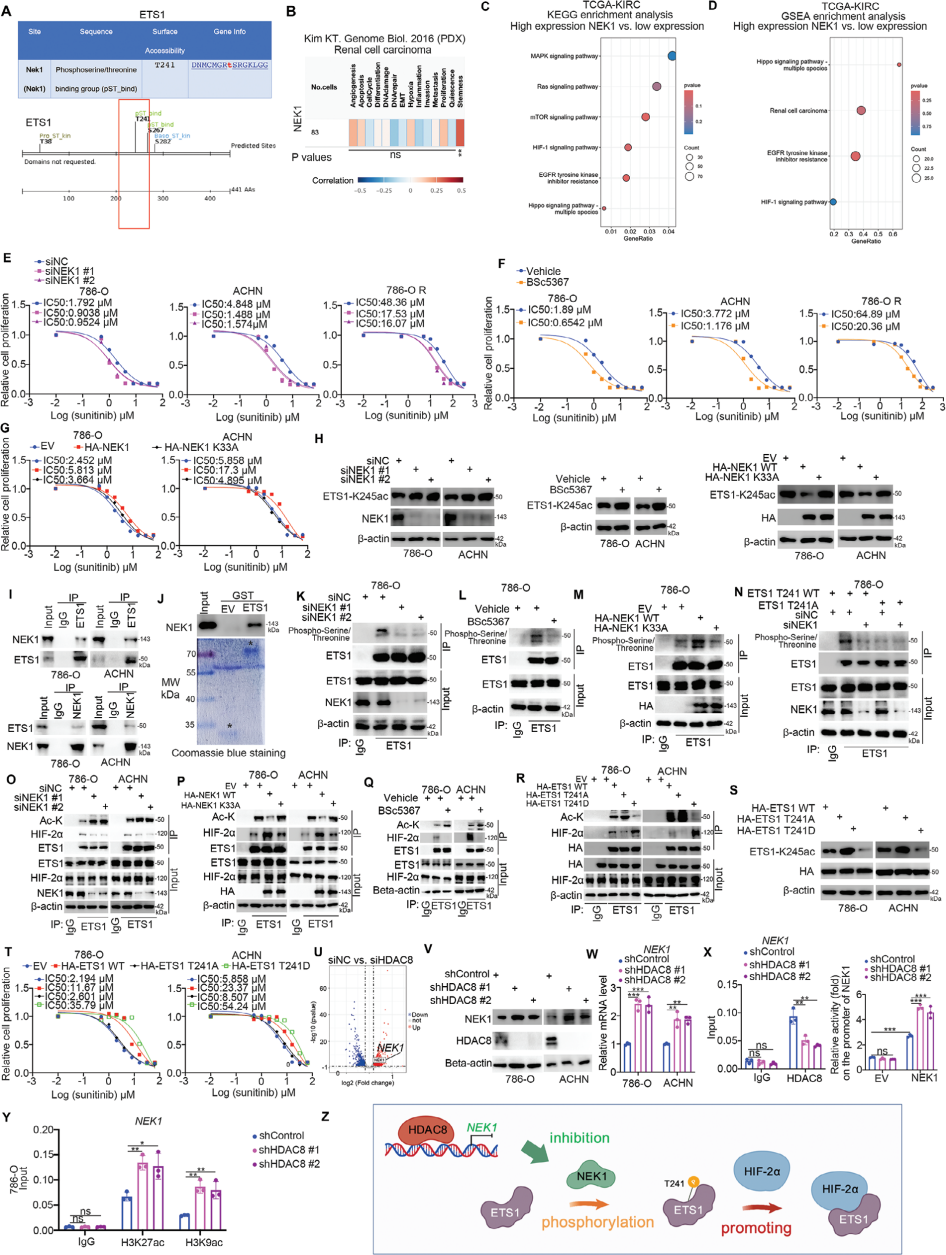
NEK1, repressed by HDAC8, phosphorylates ETS1 to enhance the binding between ETS1 and HIF‐2α. A) PhosphoSitePlus (www.phosphosite.org/) and Scansite (https://scansite4.mit.edu/#home) web tools were used to predict the ETS1‐T241 site phosphorylated by NEK1. B) The relationship between NEK1 and various tumor cell functions can be viewed through the cancerSEA website. C and D) Tumor samples in TCGA‐KIRC were grouped according to the median expression of NEK1, followed by KEGG analysis (C) and GSEA analysis (D). E–G) 786‐O, ACHN, and 786‐O R cells were transfected with indicated siRNAs for 48 h (E), or treated with vehicle or 10 µM BSc5367 for 24 h (F), or transfected with indicated plasmids for 48 h (G). Cells were collected and treated with different concentrations of sunitinib, followed by CCK8 detection. H) 786‐O and ACHN cells were transfected with indicated siRNAs for 48 h, treated with vehicle or 10 µM BSc5367 for 24 h, or transfected with indicated plasmids for 48 h. Cells were collected for western blot analysis. I and J) Co‐IP assay (I) and GST‐pulldown assay (J) were used to verify the binding between ETS1 and NEK1. K–N) 786‐O cells were transfected with indicated siRNAs for 48 h (K), treated with vehicle or 10 µM BSc5367 for 24 h (L), or transfected with indicated constructs for 48 h (M and N). Cells were collected for co‐IP and western blot analysis. O–R) 786‐O and ACHN cells were transfected with indicated siRNAs (O) or plasmids (P) for 48 h, or treated with vehicle or 10 µM BSc5367 for 24 h (Q), or transfected with indicated constructs for 48 h (R). Cells were collected for co‐IP and western blot analysis. S) 786‐O and ACHN cells were transfected with indicated plasmids for 48 h, and cells were collected for western blot analysis. T) 786‐O and ACHN cells were transfected with indicated plasmids for 48 h, Cells were collected and treated with different concentrations of sunitinib, followed by CCK8 detection. U) the Volcano plot of the RNA‐seq after knockdown of HDAC8 in 786‐O cells. V–X) 786‐O and ACHN cells were transfected with indicated shRNAs for 72 h. Cells were collected for western blot analysis (V), RT‐qPCR analysis (W), ChIP‐qPCR, and a dual‐luciferase reporter gene experiment (X). Data were presented as mean ± SD, 3 replicates. ***p < 0.001; **p < 0.01; ns, not significant. Y) 786‐O cells were transfected with indicated shRNAs for 72 h. Cells were harvested for ChIP‐qPCR assay by using IgG, H3K27ac, or H3K9ac antibodies. Data was expressed as mean ± SD with 3 replicates. **p < 0.01; *p < 0.05; ns, not significant. Z) A model depicting that HDAC8 transcriptionally represses NEK1 and NEK1 phosphorylates ETS1 at T241 site to promote ETS1 binding with HIF‐2α.

The ability of sunitinib in combination with HDAC8 knockdown or HDAC8 inhibitor treatment to inhibit xenograft tumor growth was diminished after 13 days (Figure 1). This phenomenon suggested the possible molecular mechanism of acquired drug resistance. Since the above results indicated that acetylation of ETS1 at the K245 site was important for modulating the sensitivity of ccRCC to sunitinib (Figures 4 and 5), we were curious about whether there are other posttranslational modifications around the ETS1‐K245 site that are involved in modulating the acetylation of ETS1‐K245 or the sensitivity of ccRCC to sunitinib. Analysis of the PhosphoSitePlus dataset and Scansite 4.0 web tool revealed phosphorylation at the ETS‐T241 site, and this modification might be mediated by Never in mitosis gene A (NIMA)‐related kinase 1 (NEK1) (**Figure** **6**A). The CancerSEA web tool indicated that NEK expression was positively correlated with stemness (*p* < 0.05), angiogenesis (*p* < 0.05), and hypoxia (*p* < 0.05) (Figure 6B). KEGG and GSEA enrichment analysis of the TCGA‐KIRC cohort, which was divided into 2 groups according to the expression of NEK1, indicated that high expression of NEK1 was positively associated with the MAPK, RAS, mTOR, HIF‐1, EGFR TKI resistance and Hippo signaling pathways (Figure 6C,D). Then, we demonstrated that NEK1 knockdown or treatment with an NEK1‐specific inhibitor (BSc5367) sensitized ccRCC cells to sunitinib (Figure 6E,F). In contrast, the overexpression of the wild‐type NEK1 construct but not the kinase‐dead mutant (K33A) construct increased the IC50 of sunitinib in ccRCC cells (Figure 6G). Interestingly, we found that knocking down NEK1 or treating ccRCC cells with the NEK1 inhibitor increased the acetylation level of ETS1‐K245, but overexpressing NEK1 WT decreased the level of ETS1‐K245ac (Figure 6H). Moreover, co‐IP assays indicated that NEK1 is bound to ETS1 in ccRCC cells (Figure 6I). A GST pull‐down assay also showed that ETS1 directly interacted with NEK1 (Figure 6J). Furthermore, we demonstrated that inhibition of NEK1 decreased the phosphorylation of ETS1 in 786‐O cells (Figure 6K,L). However, overexpression of NEK1 WT but not NEK K33A increased the phosphorylation level of ETS1 in 786‐O cells (Figure 6M). We subsequently showed that knockdown of NEK1 decreased the phosphorylation level of wild‐type ETS1 but had no overt effect on the phosphorylation of the ETS1 T241A mutant, which suggested that NEK1 was responsible for the phosphorylation of ETS1 at the T241 site (Figure 6N). In addition, we showed that the phosphorylation of ETS1 at the T241 site induced by NEK1 decreased the ETS‐K245ac level to enhance the interaction between ETS1 and HIF‐2α and regulate the sensitivity of ccRCC cells to sunitinib (Figure 6O–T). Importantly, we reanalyzed the RNA‐seq data after the knockdown of HDAC8 or ETS1 and found that the depletion of HDAC8 but not ETS1 increased the expression of NEK1 (Figure 6U). Subsequent RT‐qPCR analysis also showed that knockdown of HDAC8 increased the expression level of NEK1 in ccRCC cells, but inhibition of ETS1 had no effect on the expression of NEK1 (Figure 6V,W; Figure S5A, Supporting Information). It has been reported that the inhibition of HDAC8 increases the level of H3K27ac or H3K9ac, which promotes downstream gene expression in cells.^[^ ^40^, ^41^ ^]^ ChIP‐seq and ChIP‐qPCR revealed that HDAC8, H3K27ac, and H3K9ac marks were enriched at a common site in the promoter region of ETS1 (Figure 6X,Y; Figure S5B, Supporting Information). In addition, the knockdown of HDAC8 increased the binding of H3K27ac and H3K9ac to the promoter region of NEK1 and increased the transcriptional activity of NEK1 in 786‐O cells (Figure 6X,Y; Figure S5B, Supporting Information). Taken together, the above data suggest that inhibition of HDAC8 results in upregulation of NEK1 to increase the phosphorylation of ETS1 at the T241 site, which influences ETS1‐K245ac to enhance the interaction between ETS1 and HIF‐2α and is responsible for acquired drug resistance after sunitinib treatment in ccRCC cells (Figure 6Z).

### 2.7 TKI Treatment Represses the Activity of STAT3 to Upregulate HDAC8 in ccRCC

**Figure 7:**
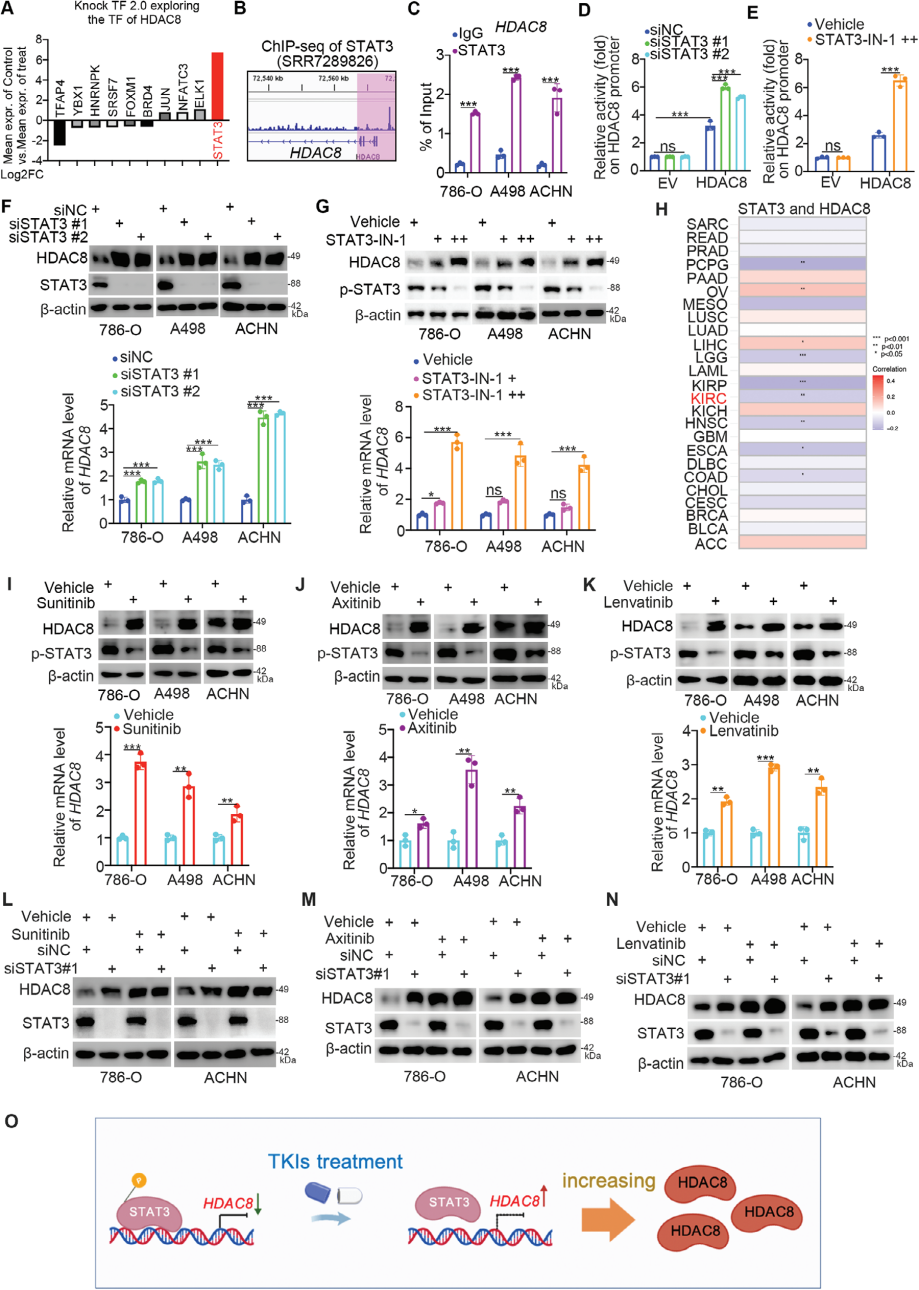
TKIs treatment represses the activity of STAT3 to up‐regulate HDAC8 in ccRCC. A and B) Knock TF2.0 website and CHIP‐seq data were used to find transcription factors of HDAC8. C) Chip‐qPCR experiments with IgG or STAT3 antibodies in 786‐O, ACHN, and A498 cell lines. Data were expressed as mean ± SD, 3 replicates. ***p < 0.001. D and E) 786‐O cells were transfected with indicated siRNAs (D) or treated with vehicle or STAT3 inhibitors (STAT3‐IN‐1, ++, 10 µM) (E), and cells were collected for dual‐luciferase reporter gene assay. Data were expressed as mean ± SD, 3 replicates. Ns, not significant; ***p < 0.001. F and G) 786‐O, ACHN, and A498 cell lines were transfected with indicates siRNAs for 48 h (F), or treated with vehicle or STAT3‐IN‐1 (+, 2 µM; ++, 10 µM) for 24 h (G). Cells were harvested for western blot analysis and RT‐qPCR analysis. Data were expressed as mean ± SD, 3 replicates. ***p < 0.001; *p < 0.05; ns, not significant. H) TCGA data were used to analyze the correlation between the expression levels of STAT3 and HDAC8 in pan‐cancer. The *p* values were indicated. I–K) 786‐O, ACHN, and ACHN cells were treated with vehicle, 5 µM sunitinib (I), 10 µM axitinib, (J) or 10 µM lenvatinib (K) for 24 h. cells were collected for western blot analysis and RT‐qPCR analysis. Data were expressed as mean ± SD, 3 replicates. ***p < 0.001; **p < 0.01; *p < 0.05. L‐N, 786‐O, and ACHN cells were transfected with siNC or siSTAT3 for 48 h, and then treated with vehicle, 5 µM sunitinib (L), 10 µM axitinib (M) or 10 µM lenvatinib (N) for another 24 h, the cells were collected for western blot analysis. O) A model depicting that TKI treatment induces HDAC8 upregulation via a STAT3‐mediated mechanism.

We noticed that sunitinib treatment increased the protein level of HDAC8, and the sunitinib‐resistant ccRCC cells and patient samples had higher expression levels of HDAC8 than did the sunitinib‐sensitive cells (Figure 1J–M). These data indicated that the upregulation of HDAC8 could be another reason for sunitinib resistance in ccRCC cells after sunitinib treatment. Given that the mRNA level of HDAC8 was also greater in the sunitinib‐resistant group than in the sunitinib‐sensitive group, we applied the knockTF‐Search web tool (www.licpathway.net) to explore the transcription factor of HDAC8 and further elucidate the potential mechanism by which HDAC8 is upregulated after sunitinib treatment. We demonstrated that STAT3 was most strongly negatively correlated with HDAC8 (**Figure** **7**A). ChIP‐seq and subsequent ChIP‐qPCR indicated that STAT3 was enriched at the promoter region of HDAC8 in ccRCC cells (Figure 7B,C). Dual‐luciferase assays and subsequent western blot and RT‐qPCR analyses also showed that STAT3 knockdown or STAT3 inhibition increased the expression of HDAC8 in ccRCC cells (Figure 7D–G). Moreover, analysis of the TCGA dataset demonstrated that the expression level of STAT3 was negatively correlated with that of HDAC8 in most solid tumors, including ccRCC (Figure 7H). These data suggest that STAT3 is a negative transcriptional regulator of HDAC8. It is well known that sunitinib inhibits the phosphorylation of STAT3 in cells.^[^ ^42^, ^43^ ^]^ Similarly, we revealed that sunitinib, axitinib, and lenvatinib treatment suppressed the phosphorylation of STAT3 and increased the expression of HDAC8 in ccRCC cells (Figure 7I–K). Next, we showed that the depletion of STAT3 attenuated the changes in HDAC8 expression after TKI treatment (Figure 7L–N). Taken together, our results indicate that TKI treatment induces HDAC8 upregulation via a STAT3‐mediated mechanism (Figure 7).

### 2.8 Construction of HDAC8‐in‐PROTAC Complexes to Enhance the Sensitivity of ccRCC to TKIs

**Figure 8:**
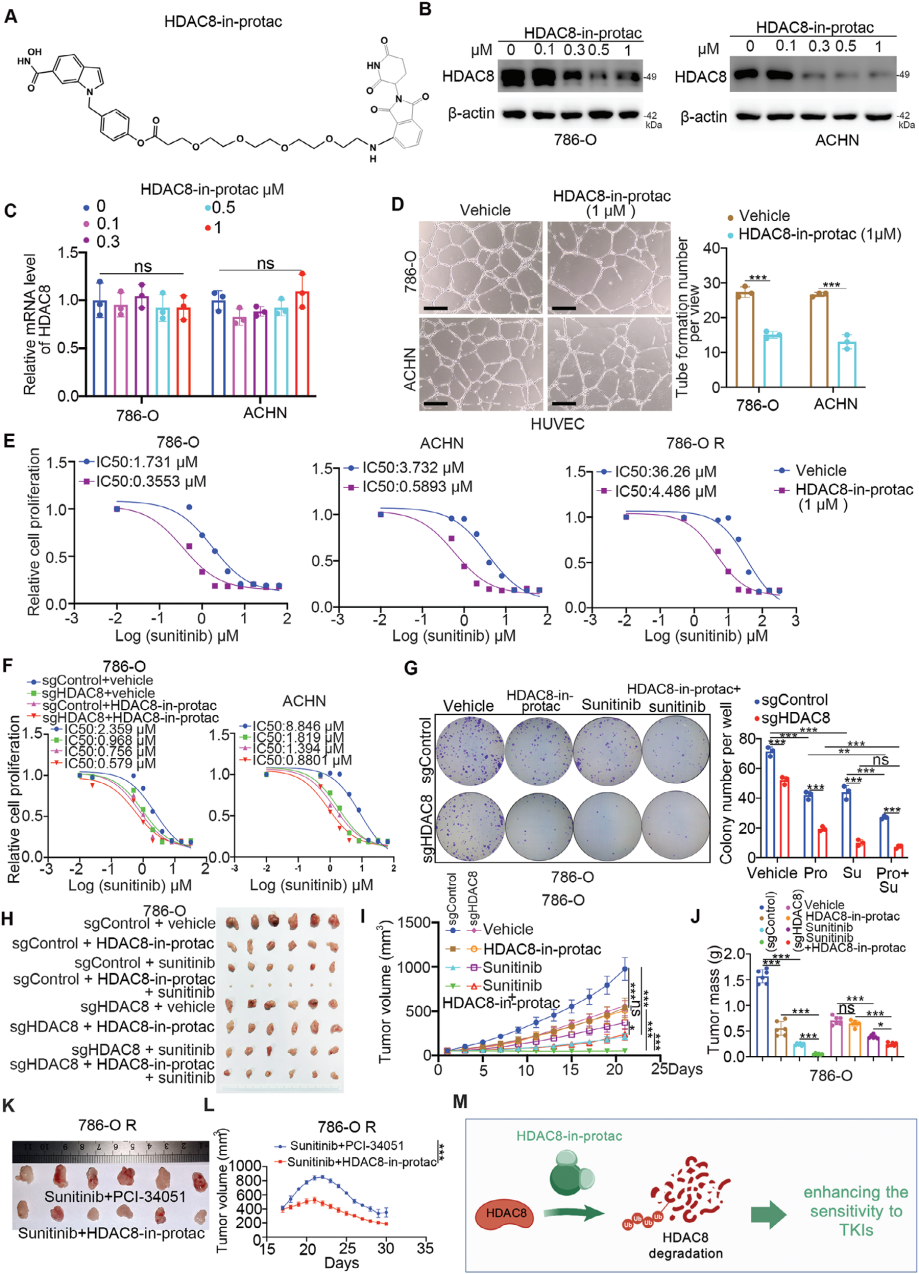
Construct HDAC8‐in‐protac to enhance the sensitivity of ccRCC to TKIs. A) Schematic representation of the HDAC8‐in‐PROTAC small molecule. B and C) 786‐O and ACHN cells were treated with different doses of HDAC8‐in‐PROTAC for 48 h, and cells were harvested for western blot analysis (B) and RT‐qPCR analysis (C). Data were expressed as mean ± SD, 3 replicates. Ns, not significant. D) 786‐O and ACHN cells were treated with vehicle or 1 µM HDAC8‐in‐PROTAC for 48 h, and cells were subjected to tube formation assay. Data were expressed as mean ± SD, 3 replicates. ***, P < 0.001. E) 786‐O, ACHN, and 786‐O R cells were treated with vehicle or 1 µM HDAC8‐in‐PROTAC for 48 h. Cells were collected and treated with different concentrations of sunitinib, followed by CCK‐8 detection. F) 786‐O and ACHN cells were transfected with indicated sgRNAs for 48 h, and these cells were treated with vehicle or 1 µM HDAC8‐in‐PROTAC for another 24 h. Cells were collected and treated with different concentrations of sunitinib, followed by CCK8 detection. G) 786‐O and ACHN cells were transfected with indicated sgRNAs for 48 h. these cells were treated with vehicle, 1 µM HDAC8‐in‐PROTAC (Pro), 5 µM sunitinib (Su), or 1 µM HDAC8‐in‐PROTAC + 5 µM sunitinib and subjected to colony formation assay. Data were expressed as mean ± SD, 3 replicates. Ns, not significant; **p < 0.01; ***p < 0.001. H–J) 786‐O cells were transfected with indicated sgRNAs for 48 h. Cells were subjected to xenograft assay. The mice were treated with vehicle, HDAC8‐in‐PROTAC (10 mg Kg^−1^, intraperitoneal injection, every day for 6 days), or sunitinib (80 mg Kg^−1^, oral administration, every day for 6 days). The image of the tumor was shown in panel H. The tumor growth curve was indicated in panel I. The tumor mass was demonstrated as in panel J. Data were expressed as mean ± SD with 6 replicates. ***p < 0.001; **p < 0.01; *p < 0.05, Ns, not significant. K and L) 786‐O R cells were subjected to xenograft assay. After the tumor volumes reached 200 mm^3^. The mice were treated with HDAC8‐in‐PROTAC (10 mg/Kg, intraperitoneal injection, every day for 10 days), or sunitinib (80 mg Kg^−1^, oral administration, every day for 10 days). The image of the tumor was shown in panel K. The tumor growth curve was demonstrated as in panel L. M) A model depicting the construct HDAC8‐in‐protic to promote the degradation of HDAC8 and enhance the sensitivity of ccRCC to TKIs.

The observation that TKI treatment increased the expression of HDAC8 in ccRCC cells could explain why the antitumor effect of the combination of HDAC8 inhibitor and sunitinib treatment was attenuated after 13 days of xenograft growth (Figure 1). According to the chemical structure of the HDAC8 inhibitor (PCI‐34051), we synthesized proteolysis‐targeting chimeras (PROTACs) of HDAC8—HDAC8‐in‐PROTACs—to decrease the protein level of HDAC8 in ccRCC cells (**Figure** **8**A–C). We subsequently showed that HDAC8‐in‐PROTACs inhibited angiogenesis in vitro (Figure 8D). Similarly, treatment with HDAC8‐in‐PROTACs decreased the IC50 values of TKIs in ccRCC cells (Figure 8E; Figure S6A,B, Supporting Information). Moreover, we demonstrated that HDAC8‐in‐PROTACs enhanced the tumor growth‐inhibiting effect of sunitinib in vivo and in vitro (Figure 8F–J). Importantly, the antitumor effect of the combination of HDAC8‐in‐PROTACs and sunitinib was greater than that of the combination of PCI‐34051 and sunitinib in 786‐O sunitinib‐resistant cell (786‐O R)‐derived xenografts (Figure 8K,L). We also revealed that the safety of HDAC8‐in‐PROTACs was similar to that of PCI‐34051 in mice (Figure S6C, Supporting Information). Taken together, these results indicate that the constructed HDAC8‐in‐PROTAC optimizes the effects of HDAC8 inhibitors and enhances the sensitivity of ccRCC to TKIs (Figure 8M).

## 3 Discussion

The field of kidney cancer treatment has fundamentally changed over the past decade. Following the cytokine era, targeted therapy has changed the treatment landscape of advanced ccRCC and has become the standard treatment option for ccRCC.^[^ ^44^ ^]^ Angiogenesis is a key process in the progression of advanced renal cell carcinoma.^[^ ^44^ ^]^ The introduction of therapeutic drugs targeting VEGF signaling, especially multitarget tyrosine kinase inhibitors (TKIs), has become a major breakthrough in the treatment of ccRCC.^[^ ^45^ ^]^ However, resistance after antiangiogenic therapy is difficult to avoid, and the high likelihood of disease progression remains a challenge. In addition to the VEGF pathway, the AKT/mTOR pathway has also been identified as a promising therapeutic target for the treatment of advanced RCC, and 2 mTOR inhibitors, everolimus and sirolimus, have received market approval.^[^ ^46^ ^]^ Immune checkpoint (PD‐1/PD‐L1/CTLA4) inhibitors have recently emerged as effective treatments for advanced RCC that prevent tumor cell immune escape. Studies have shown that targeted therapy can significantly enhance immune cell function by mechanisms such as promoting T‐cell invasion of tumors and increasing tumor cell antigenicity.^[^ ^47^ ^]^ Multiple clinical studies have shown that targeted therapy combined with nivolumab (a PD‐1 checkpoint inhibitor) has good clinical efficacy.^[^ ^48^, ^49^ ^]^ These findings set the stage for trials combining targeted antiangiogenic therapies with a new generation of immunotherapies to exploit possible synergistic effects.

HDAC8 plays a multifunctional role in cancer progression by acting on both histone and nonhistone substrates.^[^ ^29^ ^]^ Many studies support the tumorigenic effect of HDAC8, and overexpression of HDAC8 has been found in various cancers, such as gastric cancer, liver cancer, and acute lymphoblastic leukemia.^[^ ^44^ ^]^ Elimination or pharmacological inhibition of the HDAC8 gene can suppress cell proliferation, inhibit colony formation, and induce cell cycle arrest in cancer.^[^ ^29^ ^]^ HDAC8 has been implicated in a variety of immune escape strategies. Therapeutic inhibition of HDAC8 by PCI‐34051 can transform glioma cells into proinflammatory/antitumor phenotypes.^[^ ^50^ ^]^ In addition, Wang et al. showed that HDAC8 inhibited the antitumor immunity of melanoma cells by reducing the expression of PD‐L1.^[^ ^51^ ^]^ Yang et al. reported that selective inhibition of HDAC8 changes the epigenetic landscape of hepatocellular cancer cells, producing immunostimulatory signatures that work synergistically with anti‐PD‐L1 therapies to enhance T‐cell‐mediated antitumor responses.^[^ ^52^ ^]^ The ability of HDAC8 to inhibit the transformation of the noninflammatory tumor microenvironment (TME) into a T‐cell inflammatory TME should further accelerate the development of HDAC8‐specific inhibitors for cancer immunotherapy. Previous studies have shown that HDAC8 is involved in the resistance of multiple tumors to drugs. HDAC8 promotes paclitaxel resistance in breast cancer by activating TGF‐β signaling through the SIRT7‐SMAD4 axis.^[^ ^53^ ^]^ In acute myeloid leukemia (AML), HDAC8 promotes resistance to daunorubicin (DNR) by regulating the expression of IL‐6 and IL‐8.^[^ ^54^ ^]^ In AML with an internal tandem duplication (ITD) mutation of FMS‐like receptor tyrosine kinase‐3 (FLT3), the use of FLT3 tyrosine kinase inhibitors induces FOXO1‐ and FOXO3‐associated upregulation of HDAC8, thereby inactivating p53 and leading to TKI resistance.^[^ ^55^ ^]^ Here, we further investigated the role of HDAC8 in ccRCC. We demonstrated that the inhibition of HDAC8 enhanced the antitumor efficacy of TKIs in ccRCC. These findings combined with the previous findings indicate that HDAC8 is an ideal target for improving the response to both antiangiogenic targeted therapy and immune checkpoint therapy (specifically anti‐PD‐L1 therapy) in ccRCC.

ETS1 is a transcription factor that acts as a major driver of critical events that occur in advanced cancers and is strongly implicated in the progression of almost all cancers. In many cancers, including breast, colorectal, hepatocellular, and lung cancer cells, higher ETS1 protein or RNA expression is associated with higher grade, poorer differentiation, and/or increased aggressiveness.^[^ ^56^ ^]^ Previous studies have shown that ETS1 expression is associated with increased microvascular density in tumors.^[^ ^37^ ^]^ There is growing evidence that ETS1 is involved in drug resistance in cancer cells. ETS1 may lead to the development of drug resistance by upregulating the expression of the MDR1 protein, and silencing ETS1 in Adriamycin‐resistant breast cancer cells or paclitaxel‐resistant prostate cancer cells decreases the expression of MDR1 and increases drug sensitivity.^[^ ^57^, ^58^ ^]^ In addition, ETS1 may induce drug resistance by inhibiting the apoptosis of triple‐negative breast cancer cells through the promotion of the expression of αB‐crystallin (a small heat shock protein) and the chemokine receptor CXCR4.^[^ ^59^, ^60^ ^]^ ETS1 is known to be a hypoxia‐related gene that promotes progression and is associated with poor prognosis in ccRCC patients.^[^ ^61^, ^62^ ^]^ Our group has previously reported that CBX7 represses the expression of ETS1 to inactivate the TNF pathway and modulate the sensitivity of ccRCC to TKIs.^[^ ^63^ ^]^ In addition, HIF‐2α acts as a transcription factor that binds to the exon VII domain of ETS1 to play an important role in angiogenesis.^[^ ^64^ ^]^ Here, we showed that HDAC8‐induced deacetylation of ETS1 at K245 enhances the interaction between HIF–2α and ETS1 in ccRCC cells. However, Tetsu et al. reported that EGFR inhibition in lung cancer cells inactivates ETS1 function by reducing AKT activity, and the sustained inactivation of ETS1 reduces the expression of DUSP6, leading to the abnormal activation of ERK1/2 and the induction of TKI resistance.^[^ ^65^ ^]^ Thus, the specific role and associated mechanism of ETS1 in modulating the sensitivity of ccRCC to TKIs need to be further studied.

NEK1 is highly expressed in meiotic cells and is involved in DNA repair, cell cycle progression, and ciliary function regulation.^[^ ^66^ ^]^ Many studies have shown that NEK1 dysfunction is associated with the occurrence and progression of various cancers.^[^ ^66^ ^]^ Activation of the TLK1‐NEK1 axis plays a key role in the progression of prostate cancer.^[^ ^67^ ^]^ Zhu et al. showed that NEK1 is overexpressed in a variety of human glioma tissues and cell lines and is associated with advanced disease and a poor prognosis. Moreover, NEK1 knockdown can increase the sensitivity of human glioma cells to temozolomide.^[^ ^68^ ^]^ Chen et al. reported that NEK1 is highly expressed in RCC cells and exerts antiapoptotic effects through VDAC1 phosphorylation, resulting in decreased sensitivity to DNA damage treatment.^[^ ^69^ ^]^ Due to the critical role of NEK1 in DNA damage repair, NEK1‐specific inhibitors may serve as potential antitumor drug targets.^[^ ^70^ ^]^ In this study, we demonstrated that HDAC8 repression increased the expression of NEK1, which phosphorylates ETS1 at the T241 site and blocks the acetylation of ETS1 at the T245 site to promote ETS1 binding with HIF2A. This effect might be one of the mechanisms of acquired resistance to TKIs in ccRCC. However, the combination of TKIs, specific HDAC8 inhibitors, and NEK1 inhibitors might produce unpredictable side effects. Here, we also showed that TKI treatment increased the expression of HDAC8 in ccRCC, which was another mechanism underlying the acquired resistance of ccRCC to TKIs. Thus, we applied PROTAC technology to optimize HDAC8 inhibitors (PCI‐34051), which not only inhibited the activity of HDAC8 but also reduced the expression of HDAC8 in ccRCC cells.

Collectively, our results revealed that HDAC8 is a key protein involved in determining the sensitivity of ccRCC to TKIs; it mediates this effect by deacetylating ETS1 at the K245 site and promoting the interaction between ETS1 and HIF‐2α and enhancing the function of this complex. However, the antitumor effect of inhibiting HDAC8 on sensitized TKI is not very satisfactory. We subsequently showed that inhibition of HDAC8 increased the expression of NEK1, which phosphorylates ETS1 at the T241 site and impedes its acetylation at the K245 site. Moreover, we also found that TKI treatment increased the expression of HDAC8 by inhibiting STAT3 phosphorylation in ccRCC cells. These 2 findings highlight a potential mechanism of acquired resistance to TKIs in ccRCC (Figure S7, Supporting Information). Finally, we synthesized HDAC8‐in‐PROTACs to optimize the effects of HDAC8 inhibitors and overcome the resistance of ccRCC to TKIs.

## 4 Experimental Section

### Cell Lines and Cell Culture

Renal cell carcinoma cell lines A498 (#CL‐0254, Procell Life Science & Technology, Wuhan, China) and 786‐O (#CL‐0010, Procell Life Science & Technology, Wuhan, China) were cultured in RPMI‐1640 medium (Gibco, USA) supplemented with 10% fetal bovine serum (#FBS‐CP500, NEWZERUM Ltd, New Zealand) and 1% Penicillin‐Streptomycin Solution (#BL505A, Beijing Biological Sharp, China), and ACHN (#CL‐0021, Procell Life Science & Technology, Wuhan, China) were cultured in MEM medium (Gibco, USA) supplemented with 10% fetal bovine serum and 1% Penicillin‐Streptomycin Solution. All of cell lines were identified by short tandem repeat (STR) profiling in Procell Life Science & Technology and incubated at 37 °C and 5% CO_2_. Plasmocin‐ Mycoplasma Elimination Reagent (#ant‐mpp, InvivoGen) was added to the cell culture medium to prevent Mycoplasma contamination. Mycoplasma contamination was examined every 3 months by using the PlasmoTest‐Mycoplasma Detection Kit (#rep‐pt1, InvivoGen, distributor MingRui Biotech Co., LTD).

### Chemicals and Reagents

HDAC8 inhibitors, PCI‐34051 (#S2012, Selleck, China); NEK1 inhibitors, Bsc5367(#HY‐144425, MCE, China); Sunitinib (#S7781, Selleck, China); Axitinib (#S1005, Selleck, China); Lenvatinib (#S1164, Selleck, China). The siRNAs were obtained from RiboBio (Guangzhou, China). The shRNAs and plasmids were purchased from GeneCopoeia (USA). sgRNAs were designed according to https://www.synthego.com. Then sgRNAs were cloned into the lenti‐CRISPR v2 vector (Addgene, #52 961). The sequence of siRNA, shRNA, and sgRNA are shown in Table S1 (Supporting Information). Primary antibodies used are as follows: Cleaved caspased3 (#25128‐1‐AP, Proteintech, 1:500 dilution); H3K27ac (#25128‐1‐AP, Proteintech, 1:500 dilution); HDAC8 (#17548‐1‐AP, Proteintech, 1:2000 dilution); ETS1 (#12118‐1‐AP, Proteintech, 1:2000 dilution); ETS1‐K245ac (#TP40920, HUABIO, China, 1:3000 dilution); HIF‐2α (#26422‐1‐AP, Proteintech, 1:2000 dilution); NEK1 (#27146‐1‐AP, Proteintech, 1:2000 dilution); STAT3 (#10253‐2‐AP, Proteintech, 1:2000 dilution); p‐STAT3 (#ab267373, Abcam, 1:1000 dilution) and β‐actin (#20536‐1‐AP, Proteintech, 1:2000 dilution).

### Coimmunoprecipitation (co‐IP) and Western Blot Analysis

For co‐IP, cells given different treatments were collected and total protein was extracted by adding RIPA lysate supplemented with the above‐mentioned protease inhibitor and phosphatase inhibitor. After centrifugation, the supernatant was incubated with magnetic beads and specific antibodies at 4 °C overnight. After washing 6 times on the next day, the beads were mixed with the appropriate sample buffer and boiled for 10 min. Further analysis was performed by Western blot analysis.

For western blot analysis, the total protein of cells was extracted by RIPA lysate (#G2002, Servicebio, China) added with protease inhibitor (#P1005, Beyotime, China) and phosphatase inhibitor (#P1081, Beyotime, China). After calculating the volume of different samples according to concentration which was determined by BCA method, the protein was boiled and subjected to sodium dodecyl sulfate‐polyacrylamide gel electrophoresis (SDS‐PAGE) gel separation. And then, the proteins were transferred to the 0.45 µm polyvinylidene fluoride membranes (Millipore, USA). The membranes were sealed with 5% skim milk and incubated with the primary antibody diluted by 5% BSA overnight at 4 °C. On the second day, after being washed by 1 × TBST 3 times, the membranes were incubated with the second antibody diluted by 5% BSA at room temperature for 1 h. After being washed another 3 times, the membranes and ECL luminescent solution (Thermo Fisher Scientific, USA) were imaged on ChemiDoc XRS (Bio‐Rad Laboratories, USA), and the pictures were preserved and used for follow‐up analysis.

### Glutathione S‐Transferase (GST) Pull‐Down Assay

The total protein of cells was extracted by RIPA lysate added with the above‐mentioned protease inhibitor and phosphatase inhibitor. GST fusion protein was immobilized with glutathione‐sepharose beads (GE Healthcare Life sciences, USA). The cell lysate was incubated with beads at 4 °C overnight. On the next day, the beads were washed 6 times with a binding buffer and then re‐suspended in the sample buffer. After SDS‐PAGE, the glues were dyed with Coomassie Blue Staining Solution (#G2059, Servicebio, China) for 6–8 h. After being decolorized, glues were photographed and used for follow‐up analysis.

### Quantitative Real‐Time PCR (RT‐qPCR) and Chromatin Immunoprecipitation (ChIP)‐qPCR Analysis

The total RNA was extracted by TRIzol reagent (Thermo Fisher Scientific, USA). The concentration of RNA was determined by detected by NanoDrop2000 (Thermo Fisher Scientific, USA), and the reverse transcription kit (#AG11728, Accurate Biology, Hunan, China) was used to complete the reverse transcription. Gene‐specific primers were purchased from BGI Co., Ltd (Beijing, China) and the sequence was provided in Table S1 (Supporting Information). qPCR was performed by cDNA, primers, and Evo M‐MLV one‐step RT‐qPCR kit (#AG11732, Accurate Biology, China).

As for ChIP‐qPCR analysis, the ChIP Kit Magnetic – One Step (Abcam, ab156907, USA) and chromatin extraction kit (Abcam, ab117152, USA) were used to perform the ChIP‐qPCR according to the instructions. The primer sequences for RT‐qPCR were provided in Table S2 (Supporting Information). The primer sequences for ChIP‐qPCR were provided in Table S3 (Supporting Information).

### Immunohistochemistry (IHC)

Immunohistochemical staining was carried out by using corresponding antibodies and tissue microarray slides (#U081ki01) purchased from Bioaitech, China. The antibodies used were as follows: HDAC8 (#17548‐1‐AP, Proteintech, 1:2000 dilution); CD31 (#11265‐1‐AP, Proteintech, 1:3000 dilution); ETS1‐K245ac (#TP40920, HUABIO, China, 1:2000 dilution). The staining scores of each tissue were obtained by multiplying the percentage values and staining intensity, which was divided into 3 grades: 0 for none, 1 for low, 2 for medium, and 3 for strong.

### Transient Transfection of Plasmids

Cells were planted in 10 cm^2^ culture dishes and starved with Opti‐MEM Reduced‐Serum Medium (Opti‐MEM, Gibco, USA) for 12 h before transfection. Lipo2000 and siRNA or plasmid were added to Opti‐MEM respectively to incubate for 5 min. Then, Opti‐MEM mixed with lipo2000 was transferred to Opti‐MEM containing siRNA/plasmid to incubate for another 20 min. The mixture was added to culture dishes which were washed with PBS, and then the volume was added to 4 mL with Opti‐MEM. After incubation for another 6–8 h, Opti‐MEM was replaced with a complete medium.

### Cell Proliferation Assay

CCK‐8 assay was used for the in vitro cell proliferation assay. Cells were seeded in 96‐well plates and given different treatments with 3 repetitions. 10 µL cck‐8 reagent was added to each well in the plates and then incubated at 37 °C and 5% CO_2_ for 2 h. The absorbance of cells at 450 nm was measured by a microplate reader (Thermo Fisher Scientific, USA).

### Mouse Study

BALB/C‐nu/nu mice (6 weeks old) were used in all animal experiments and purchased from SJA Experimental Animals Company (Changsha, China). All animal experiments were examined and approved by the Experimental Animal Ethics Committee of the second Xiangya Hospital of Central South University (Animal license number: 20 231 310). Our study examined male and female animals, and similar findings were reported for both sexes. Mice were raised under standard conditions, where water and food could be contacted freely, and the light / dark cycle was 12 h. The target 786‐O cells were injected subcutaneously into the left back of mice at the concentration of 5 × 10^6^ per mouse, and mice were continued to feed for a period of time. And then, mice were euthanized and tumors were removed. The length and width of the tumor were measured with a Vernier caliper, and the tumor volume was calculated according to the (L × W^2^) / 2 formula.

### Statistical Analysis

The experimental data are expressed as the mean ± standard deviation (SD) of 3 independent experiments/samples. GraphPad Prism 9 software was used to calculate the P value using unpaired two‐sided Student's t‐test to compare values between 2 groups or a one‐way analysis of variance (ANOVA) followed by Tukey's multiple comparisons post hoc test to compare values between more than 2 groups. The difference was considered to be statistically significant when *p*‐value was less than 0.05. Significance levels are: ns, not significant; *p < 0.05; **p < 0.01; ***p < 0.001.

### Ethics Approval and Consent to Participate

The study was conducted in accordance with the principles of the Declaration of Helsinki principles. It was approved by the Animal Use and Care Committees at the Second Xiangya hospital, Central South University.

## Conflict of Interest

The authors declare no conflict of interests.

## Author Contributions

K.Q., W.L., and S.R. contributed equally to this work. K.Q., W.L., and S.R. performed the methodology. W.P., B.Q., and X.L. performed formal analysis. W.X., L.Z. performed conceptualization. Y.W. and X.J. performed. Investigation. X.J. performed Project administration

## Supporting information

Supporting Information

## Data Availability

The data that support the findings of this study are available from the corresponding author upon reasonable request.
