# Supplementary material for: HDAC8 Enhances the Function of HIF‐2α by Deacetylating ETS1 to Decrease the Sensitivity of TKIs in ccRCC
Source: Adv Sci (Weinh). 2024 Jul 29;11(36):2401142. doi: 10.1002/advs.202401142 (PMC11423204; doi:10.1002/advs.202401142)
Supplement: Supplementary file 1 — Supporting Information [file ADVS-11-2401142-s001.docx]

HDAC8 enhances the function of HIF-2α by deacetylating ETS1 to decrease the sensitivity of TKIs in ccRCC

Kang Qian, Wei Li, Shangqing Ren, Weilin Peng, Bei Qing, Xinlin Liu, Xiong Wei, Liang Zhu, Yapeng Wang, Xin Jin


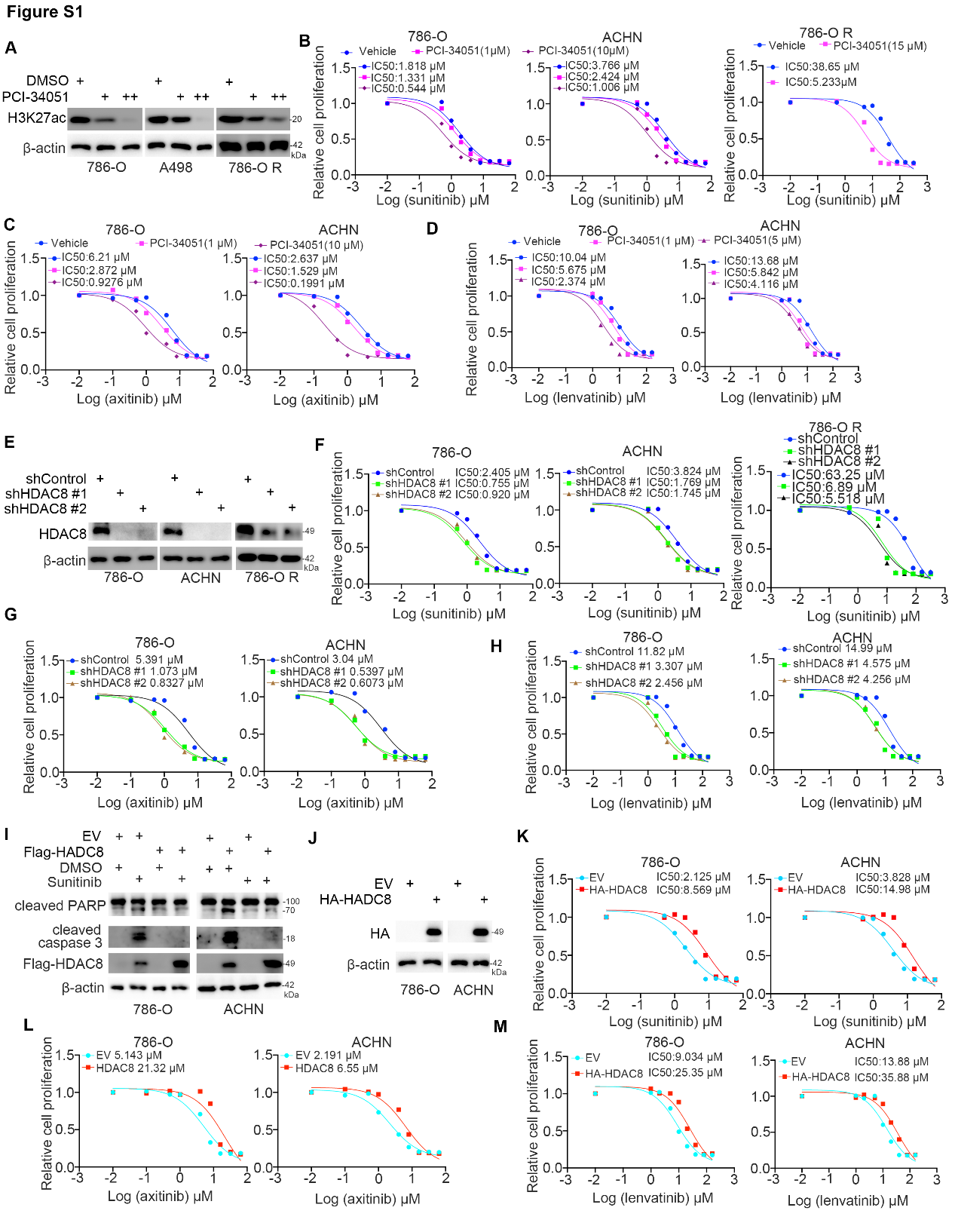


**Figure S1. A**, The cells as shown in the figure were treated with PCI-34051 (+, 1 μM; ++, 10 μM) or DMSO (vehicle) and subjected to western blot detection. **B-D**, After treating the cells shown in the figure with vehicle or different doses of PCI-34051, the cells were collected and treated with different doses of sunitinib (B), axitinib (C) or lenvatinib (D), followed by CCK8 experiments. **E-H**, 786-O, ACHN, and 786-O R cells were transfected with indicated shRNAs for 72 hours, cells were collected for western blot assay (E) and treated with different doses of sunitinib (F), axitinib (G) or lenvatinib (H) followed by CCK8 experiments. **I**, 786-O and ACHN cells were transfected with indicated plasmids for 48 hours. Then these cells were treated with or without 10 μM for another 24 hours. Cells were harvested for western blot analysis. **J-M**, 786-O and ACHN cells were transfected with indicated plasmids for 48 hours, cells were collected for western blot assay(J) and treated with different doses of sunitinib (K), axitinib (L) or lenvatinib (M) followed by CCK8 experiments.


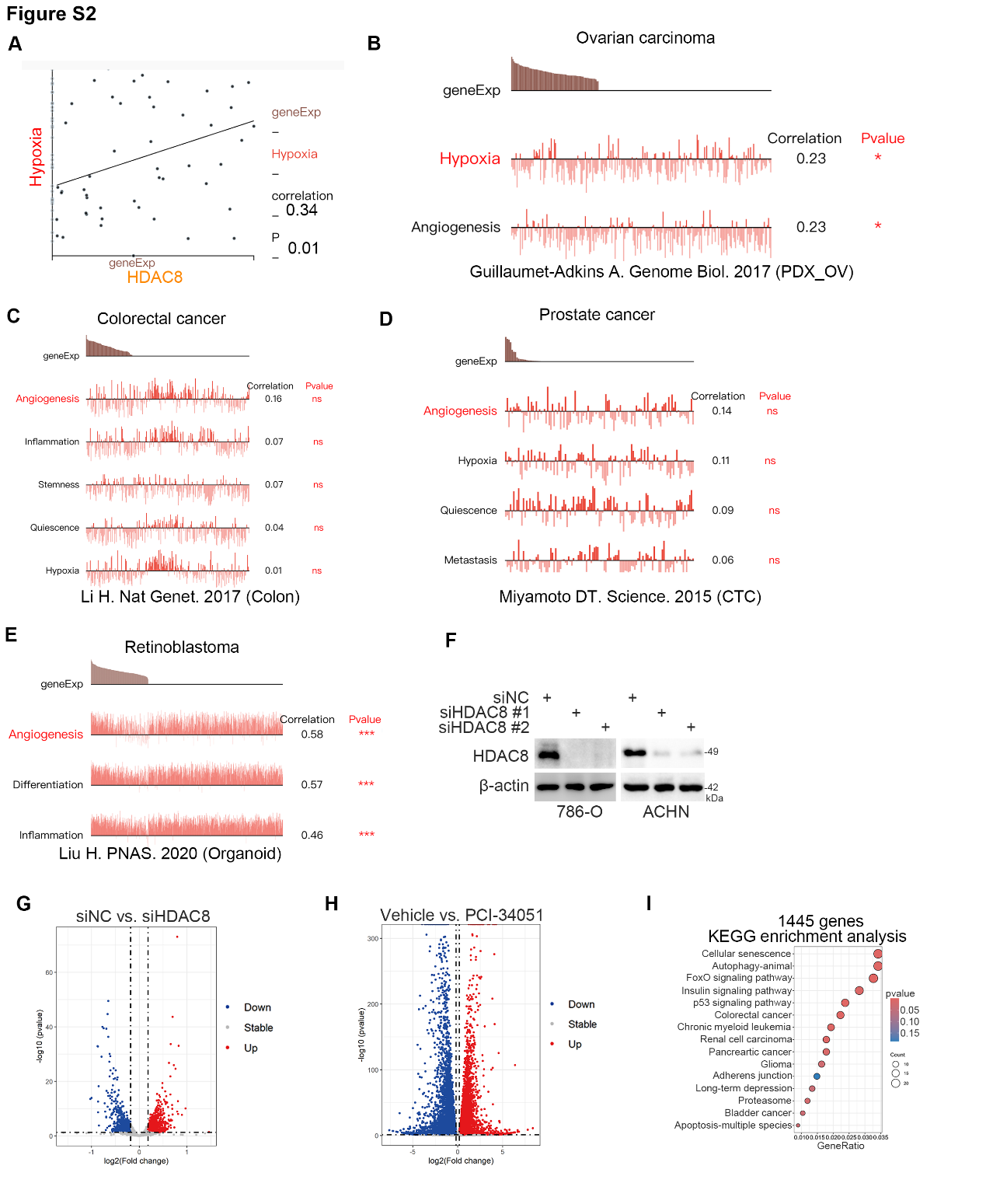


**Figure S2.** **A**, The correlation between the expression level of HDAC8 and hypoxia was queried on the cancerSEA website. **B-E**, The correlation of HDAC8 expression levels with various phenotypes in ovarian cancer (B), colon cancer (C), prostate cancer (D), and retinoblastoma (E) was queried on the cancerSEA website. **F**, 786-O and ACHN cells were transfected with indicated siRNAs for 48 hours. Cells were harvested for western blot analysis. **G and H**, the Volcano Plot of transcriptome sequencing after transfected with indicated siRNAs for 48 hours or treated with PCI-34051 for 24 hours in 786-O cells. **I**, The intersection of the changed molecules after knockdown of HDAC8 and PCI-34051 treatment was performed, KEGG enrichment analysis was performed on all the molecules with differences.


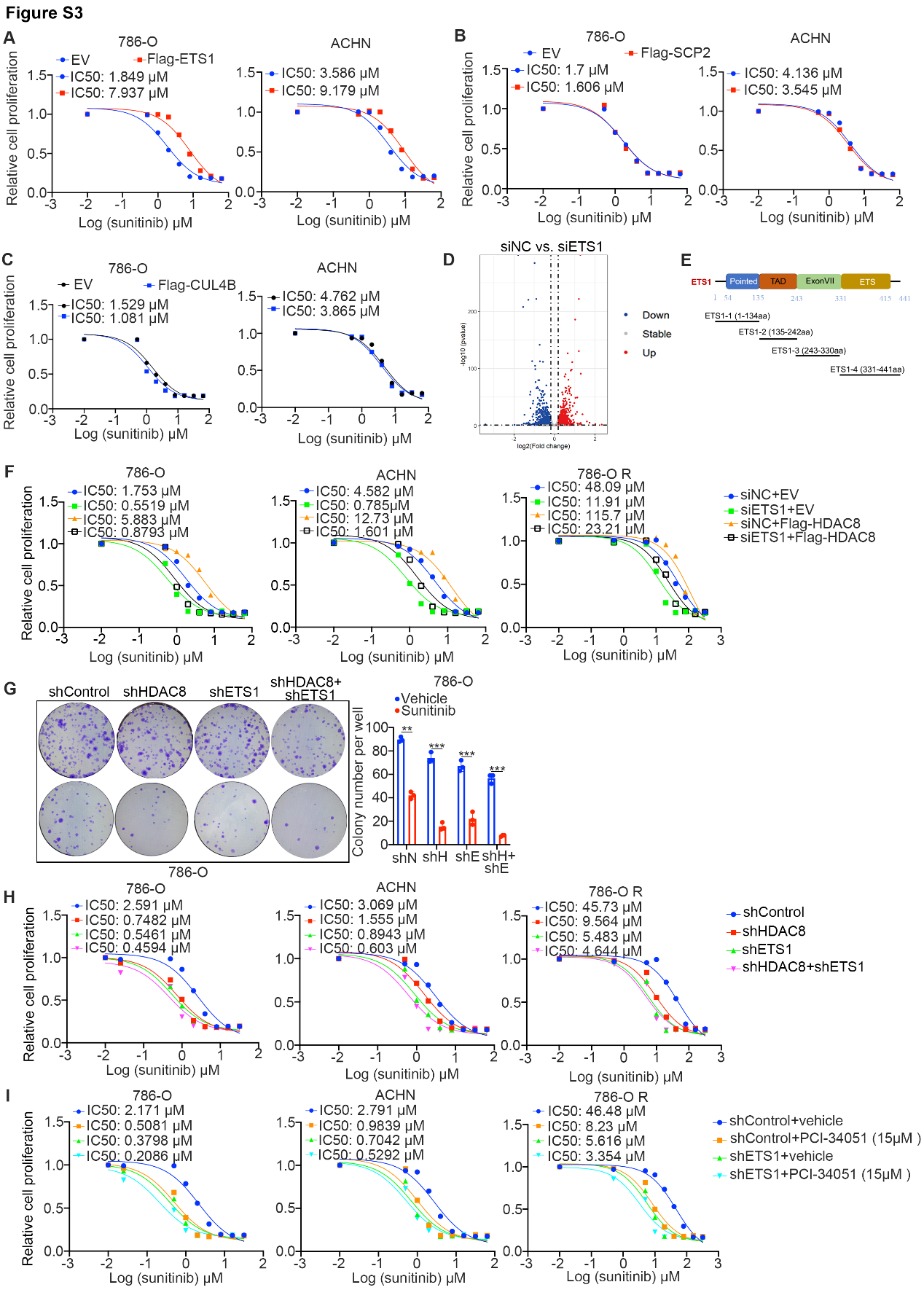


**Figure S3.** **A-C**, 786-O and ACHN cells were transfected with indicated plasmids for 48 hours. Cells were treated with a serial dose of sunitinib and subjected to CCK-8 assay. **D**, the Volcano Plot of transcriptome sequencing after transfected with indicated siRNAs for 48 hours in 786-O cells. **E**, Schematic diagram depicting a set of ETS1 recombinant protein constructs. **F**, 786-O, ACHN and 786-O R cells were transfected with indicated siRNAs or plasmids for 48 hours. Cells were treated with a serial dose of sunitinib and subjected to CCK-8 assay. **G**, 786-O cells were transfected for 48 hours as indicated in the figure, and the cells were continued to be treated with vehicle or 5 μM sunitinib for 24 hours. The cells were collected for colony formation assay. Data were expressed as mean ± SD with three replicates. ***, P<0.001; **P<0.01. **H**, 786-O, ACHN and 786-O R cells were transfected with indicated shRNAs for 72 hours. Cells were treated with a serial dose of sunitinib followed CCK-8 assay. **I**, 786-O, ACHN and 786-O R cells were transfected with indicated shRNAs for 72 hours. Cells were treated with or without 15 μM PCI-34051 and a serial dose of sunitinib followed CCK-8 assay.


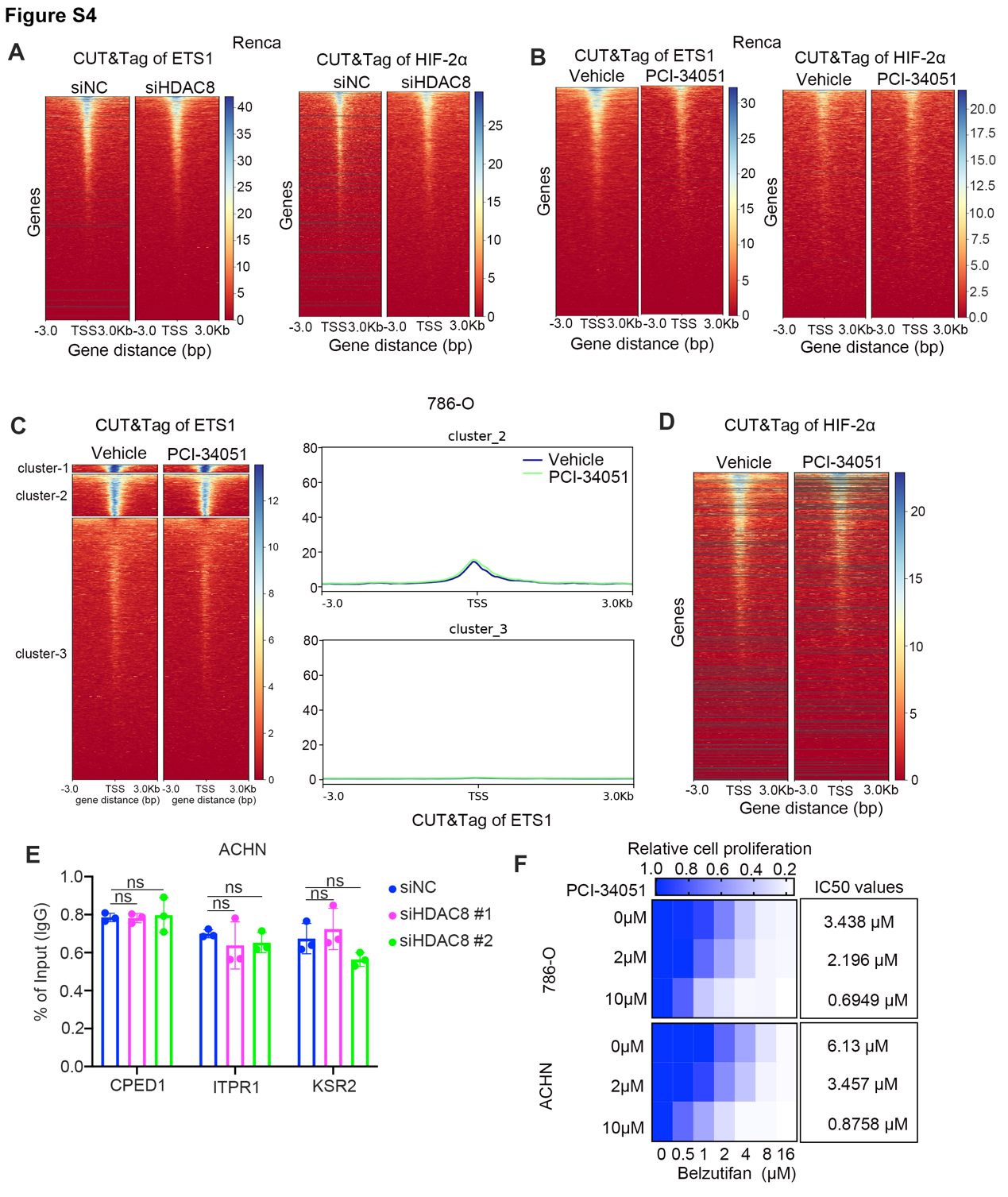


**Figure S4.** **A**, Renca cells were transfected with siNC or siHDAC8 for 48 hours, and cells were collected for CUT&Tag by using the ETS1 or HIF-2a antibodies. **B**, Renca cells were treated with vehicle or 10 μM PCI-34051 for 24 hours, and cells were collected for CUT&Tag by using the ETS1 or HIF-2a antibodies. **C and D**, 786-O cells were treated with vehicle or 10 μM PCI-34051 for 24 hours, and cells were collected for CUT&Tag by using the ETS1 (C) or HIF-2a (D) antibodies. **E**, ACHN cells were transfected with indicated siRNAs for 48 hours, cells were collected for ChIP-qPCR analysis. Data were presented as mean ± SD with three replicates. Ns, not significant. **F**, 786-O and ACHN cells were treated with vehicle or 10 μM PCI-34051 for 24 hours. Cells were treated with Belzutifan at different concentration gradients for 24 hours, and CCK8 experiments were performed to measure the IC50 values of Belzutifan.


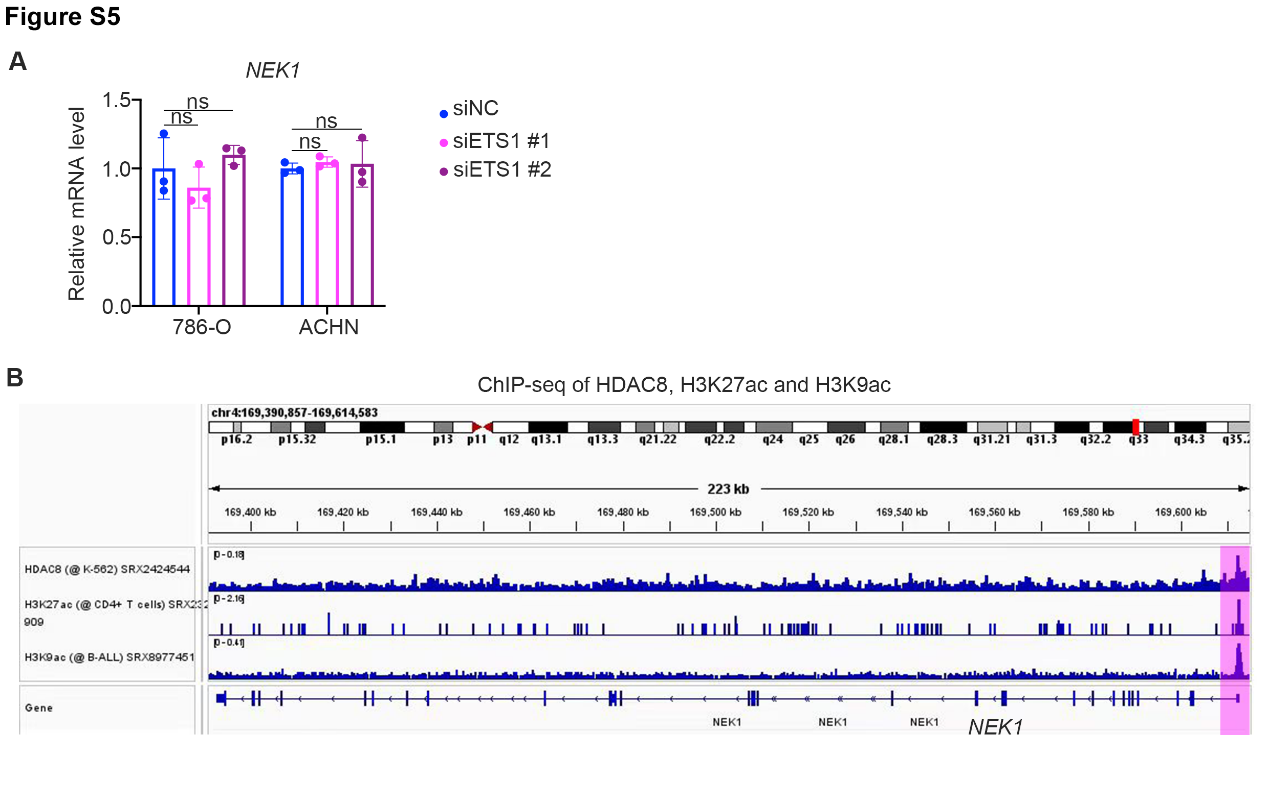


**Figure S5.** **A**, 786-O and ACHN cells were transfected with indicated siRNAs for 48 hours. Cells were collected for RT-qPCR assay. Data was expressed as mean ± SD with three replicates. ns, not significant. **B**, ChIP-seq data of HDAC8, H3K27ac, and H3K9ac were used to analyze promoter binding to NEK1.


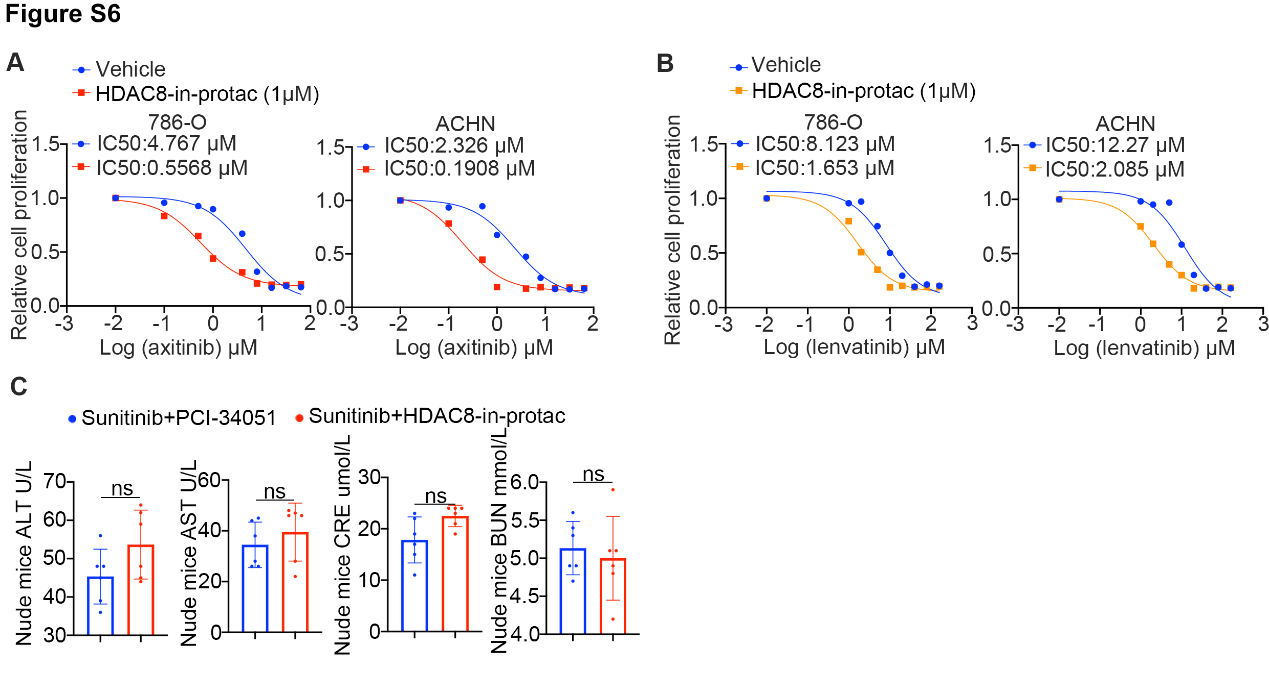


**Figure S6. A**, 786-O and ACHN cells were treated with vehicle or 1 μM HDAC8-in-PROTAC, and different doses of axitinib followed CCK8 assay. **B**, 786-O and ACHN cells were treated with vehicle or 1 μM HDAC8-in-PROTAC, and different doses of lenvatinib followed CCK8 assay. **C**, 786-O R cells were subjected for xenograft assay. After the tumor volumes reached to 200 mm3. The mice were treated with HDAC8-in-PROTAC (10mg/Kg, intraperitoneal injection, every day for 10 days), or sunitinib (80mg/Kg, oral administration, every day for 10 days). Measurements of ALT, AST, CRE, BUN were conducted in nude mice before excised the tumor. All data were shown as mean values ± SD (n = 6), ns, not significant.


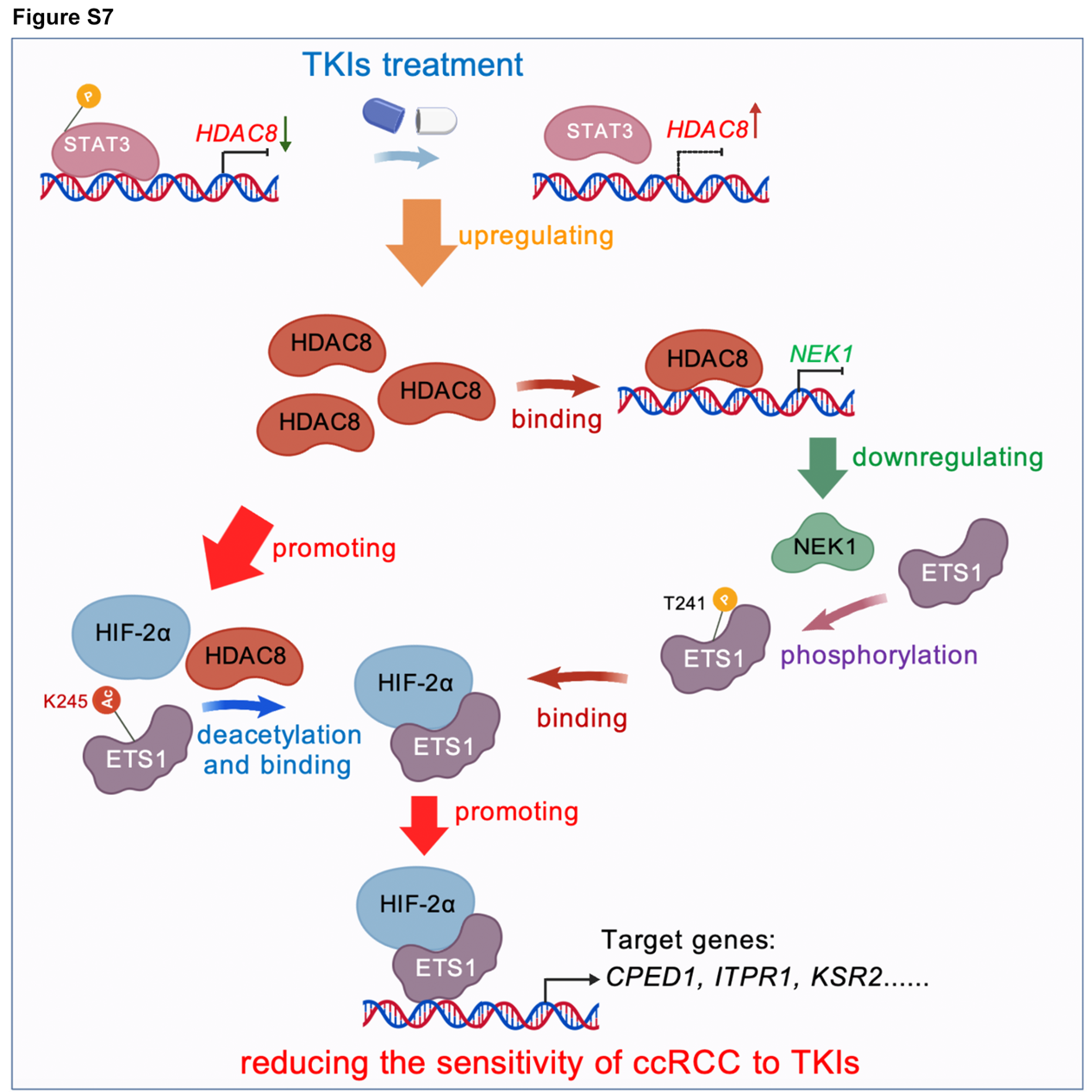


**Figure S7.** A model depicting that TKIs treatment increased the expression of HDAC8 by inhibiting STAT3 phosphorylation in ccRCC cells, the up-regulated HDAC8 deacetylated ETS1 at the K245 site to promote the interaction between ETS1 and HIF-2α complex and enhance the function of this complex, which lead to decreasing the sensitivity of ccRCC to TKIs. On the other hand, inhibition of HDAC8 increased the expression of NEK1 to phosphorylate ETS1 at the T241 site and impede its acetylation at the K245 site, which is one of reasons that the antitumor effect of inhibiting HDAC8 on sensitized TKI is not very satisfactory.

**Supplementary Material and Methods**

**Collection of clinical specimens**

Clinical specimens of ccRCC used were the same as the previously reported(1). In brief, clinical specimens of ccRCC were collected from the Department of Urology, The Second Xiangya Hospital, Central South University. Ethical approval for the use of human tissues (patients with renal cancer with or without sunitinib resistance) was obtained by the local ethics committee (The Second Xiangya Hospital, China; approval no. 2021068). Written informed consent was acquired from all patients before surgery. We collected the specimens from patients with ccRCC that were diagnosed at the late stage and underwent a palliative resection of the tumor following sunitinib therapy. Tissue specimens are collected from the primary cancer tissue. Postoperative imaging examination, such as computed tomography, was used to evaluate the therapeutic effect of sunitinib therapy. According to the RECIST version 1.1(2), we deﬁned those patients achieved complete remission or partial remission as sunitinib sensitive, and patients with progressive disease as sunitinib resistance.

**RNA sequencing and analysis**

A total of 1 µg of RNA per sample was used as the starting material for RNA sequencing (RNA-seq). RNA integrity was assessed using the RNA Nano 6000 Assay Kit of the Bioanalyzer 2100 system (Agilent Technologies, CA, USA). Clean data (clean reads) were obtained by removing reads containing adapter, reads containing ploy-N and low-quality reads from raw data. At the same time, Q20, Q30 and GC content the clean data were calculated. All the downstream analyses were based on the clean data with high quality. Sequencing libraries were generated using the NEBNext Ultra RNA Library Prep Kit for Illumina (NEB, USA) following the manufacturer’s instructions, and index codes were added to attribute sequences to each sample. Clustering of the samples was performed on the cBot Cluster Generation System using the TruSeq PE Cluster Kit v3-cBot-HS (Illumina) according to the manufacturer’s instructions. After cluster generation, libraries were sequenced on an Illumina Novaseq platform, and 150-bp paired-end reads were generated. FeatureCounts v1.5.0-p3 was used to count the read numbers mapped to each gene. Differential expression analysis was performed using the DESeq2 R package (1.16.1), and the cluster Profiler R package was used to test the statistical enrichment of differentially expressed genes (DEGs) in KEGG (Kyoto Encyclopedia of Genes and Genomes) pathways. Three replicates were performed in each group.

**CUT&Tag analysis**

CUT&Tag experiments for mapping chromatin segments onto the genome were performed by Romics Biotechnology Ltd (Shanghai, China). We performed this assay with 786-O or Renca cells with indicated antibody, according to a previous report(3). The resulting data was mapped to the hg38 genome via bowtie2. Call peaks were performed via MACS3. Visualize genome-wide normalized signal coverage traces in IGV.

**Luciferase reporter assay**

GV592-HDAC8 promoter reporter plasmids WT (- 150bp, chrX: 72576600-72575750) were constructed into GV592 backbone (MCS-SV40-firefly_luciferase-PolyA-Tk-Renila_Luciferase-PolyA) by GENECHEM (Shanghai, China). The GV592-HDAC8 promoter reporter plasmid was transfected into cells by using the Lipofectamine 2000 (Thermo Fisher Scientific, USA). Forty-eight hours post transfection, cells harvested and tested with the Dual Luciferase Reporter Gene Assay Kit (Beyotime, RG027). All experiments were performed in three times.

**Establishing the sunitinib-resistant 786-O (786-O R) cells**

A sunitinib-resistant cell model was generated by continuous treatment of 786-O cells with sunitinib treatment as reported previously (1), and the success of the construct was tested by IC50 value after about 3 months.

**Reference**

1. Sun Y, Zhu L, Liu P, Zhang H, Guo F, and Jin X. ZDHHC2-Mediated AGK Palmitoylation Activates AKT-mTOR Signaling to Reduce Sunitinib Sensitivity in Renal Cell Carcinoma. *Cancer Res.* 2023;83(12):2034-51.

2. Abu-Ghanem Y, van Thienen JV, Blank C, Aarts MJB, Jewett M, de Jong IJ, et al. Cytoreductive nephrectomy and exposure to sunitinib - a post hoc analysis of the Immediate Surgery or Surgery After Sunitinib Malate in Treating Patients With Metastatic Kidney Cancer (SURTIME) trial. *BJU Int.* 2022;130(1):68-75.

3. Kaya-Okur HS, Wu SJ, Codomo CA, Pledger ES, Bryson TD, Henikoff JG, et al. CUT&Tag for efficient epigenomic profiling of small samples and single cells. *Nat Commun.* 2019;10(1):1930.

**Table S1. The siRNA, shRNA and sgRNA sequences.**

| shHDAC8 #1 | 5'- GATCGGAAGTATACCAAGCCTTTAACTCGAGTTAAAGGCTTGGTATACTTCCTTTTTG -3' |
| --- | --- |
| shHDAC8 #2 | 5'- GATCGCAAGTGTCTTAAGTACATCCCTCGAGGGATGTACTTAAGACACTTGCTTTTTG -3' |
| shETS1 #1 | 5'- GATCGCAACTCAGGAAGTTCCTACTCTCGAGAGTAGGAACTTCCTGAGTTGCTTTTT-3' |
| shETS1 #2 | 5'- GATCGGGAAGTTCCTACTGGTCTTGACTCGAGTCAAGACCAGTAGGAACTTCCTTTTTG -3' |
| sgHDAC8 #1 | 5'- CGGAGGAACCGGCGGACAGT -3' |
| sgHDAC8 #2 | 5'- CCACTGTCCGCCGGTTCCTC -3' |
| siHDAC8 #1 | 5'- UGCUUAUGCAGUGCAUAUGCU -3' |
| siHDAC8 #2 | 5'- AACUGAAUGCGUCUUCUACAC -3' |
| siETS1 #1 | 5'- AGAACUUCUGGAAGUCUACAC -3' |
| siETS1 #2 | 5'- ACUAUCGUAGCUCUCUAUGCU -3' |
| siNEK1 #1 | 5'- AAUCUGAUCCUCUUGAAACAA -3' |
| siNEK1 #2 | 5'- UCUUUCGUUCUAGUUGUUCUU -3' |
| siSTAT3 #1 | 5'- AGUUGAAAUCAAAGUCAUCCU -3' |
| siSTAT3 #2 | 5'- AAAAAAGUUUACAUUCUUGGG -3' |

**Table S2. The primer sequences for RT-qPCR.**

| **Species** | **Gene** | **Forward (5’-3’)** | **Reverse (5’-3’)** |
| --- | --- | --- | --- |
| Human | GAPDH | AATGGGCAGCCGTTAGGAAA | GCGCCCAATACGACCAAATC |
| Human | HDAC8 | GAGGAGCAGGAACTGGAACC | AGATGCTTCATCTCTCATCTGCT |
| Human | NEK1 | CTTACCCTACCCTGGCCTCT | TGACATCCTGACACTCGTCT |
| Human | ETS1 | TAAGTGAGGTGCTGAGAGCAG | GGAGCTCTTCCGAGCTGATG |

**Table S3. The primer sequences for ChIP-qPCR.**

| Gene（Human） | Forward primer (5′ - 3′) | Reverse primer (5′ - 3′) |
| --- | --- | --- |
| ITPR1 (ETS1, HIF-2α) | CATGTGGATGTGCTGCTGAA | TAATCCCATGTCCGCGAAGA |
| CPED1 (ETS1, HIF-2α) | GCAGCCTCATTTGGGGAAA | CACACGCACACAGACACATA |
| KSR2 (ETS1, HIF-2α) | TCTGAGTCTCTGGTCCCTGA | AGCCGGTGTGTAAAGAGTGA |
| NEK1 (HDAC8, H3K27ac, H3K9ac) | CGAATGCGGACTAAGGGAGA | CCGTCTCGTCCTTTCTGTCT |
| HDAC8 (STAT3) | GCTGAATGGCTCTAAACTGGA | TGCCAATTCCAGAGCATTGT |

**Table S4. The quantitative acetylation proteomics after knockdown HDAC8 in 786-O cells**

| Gene Name | Positions within Proteins | Intensity HDA-Si-1 | Intensity HDA-Si-2 | Intensity HDA-Si-3 | Intensity HDA-NC-1 | Intensity HDA-NC-2 | Intensity HDA-NC-3 | HDA-Si/HDA-NC | t test p value | HDA-Si/HDA-NC |
| --- | --- | --- | --- | --- | --- | --- | --- | --- | --- | --- |
| EIF5A | 47;47 | 2E+06 | 3E+06 | 3E+06 | 9E+05 | 925055 | 129214 | 4.1979 | 0.0069 | 4.1979 |
| SCP2 | 546 | 3E+05 |  | 252590 |  | 102687 | 34198 | 3.8728 | 0.0327 | 3.8728 |
| CUL4B | 55 | 60762 | 68386 | 71137 | 26065 | 22138 | 15232 | 3.1574 | 0.0005 | 3.1574 |
| TUBA1B | 304;304;304;304;304;304 | 1E+05 |  | 130421 |  | 57376 | 30833 | 3.0243 | 0.0224 | 3.0243 |
| H1-4 | 17 | 2E+05 | 153320 | 192215 | 88156 | 28716 | 77165 | 2.6938 | 0.007 | 2.6938 |
| ME2 | 156 | 1E+06 | 1E+06 | 1E+06 | 6E+05 | 498448 | 368113 | 2.4979 | 0.0021 | 2.4979 |
| TCOF1 | 400 | 1E+05 | 119179 | 115208 | 39848 | 29494 | 79228 | 2.2748 | 0.0164 | 2.2748 |
| ATP5PD | 63 | 30614 | 28550 |  | 15385 | 11177 |  | 2.2274 | 0.02 | 2.2274 |
| CLIC1 | 13 | 45410 |  | 49896 | 42151 | 1768 |  | 2.17 | 0.3334 | 2.17 |
| VASP | 283 | 61101 | 70894 | 63337 | 53588 |  | 6646.3 | 2.1619 | 0.1457 | 2.1619 |
| NACA4P | 141 | 9E+05 | 1E+06 | 1E+06 |  | 555776 | 415165 | 2.1468 | 0.0073 | 2.1468 |
| ETS1 | 245 |  | 318700 | 375721 | 2E+05 |  | 138891 | 2.0979 | 0.0431 | 2.0979 |
| DSTN | 127 | 2E+05 | 302542 | 404506 | 2E+05 | 147472 |  | 2.084 | 0.0855 | 2.084 |
| RACK1 | 172 | 39690 | 28701 |  | 18308 | 6172.1 | 24818 | 2.0809 | 0.1176 | 2.0809 |
| TMSB10 | 4 | 1E+05 | 567795 | 142206 | 1E+05 | 123739 |  | 2.0733 | 0.4869 | 2.0733 |
| H1-2 | 17 | 4E+05 | 366579 | 582772 | 4E+05 | 158990 | 71034 | 2.04 | 0.1463 | 2.04 |
| CTTN | 272 | 1E+05 | 114225 |  |  | 96273 | 13877 | 1.9987 | 0.3154 | 1.9987 |
| RPS8 | 125 | 75637 | 96044 | 122602 | 46709 | 54416 | 46232 | 1.9971 | 0.0241 | 1.9971 |
| CREBBP | 1736 | 3E+05 | 233063 | 166927 | 93223 | 135685 | 106443 | 1.9823 | 0.0251 | 1.9823 |
| NAP1L4 | 146 | 42270 | 44920 | 57310 | 28835 | 19333 | 27263 | 1.9157 | 0.0138 | 1.9157 |
| CREBBP | 1741 | 2E+05 | 233063 | 152154 | 93223 | 135685 | 106443 | 1.8309 | 0.0332 | 1.8309 |
| RPL21 | 78 | 73151 | 66958 | 55551 | 31941 | 40139 |  | 1.8096 | 0.0283 | 1.8096 |
| GSPT1 | 196;325 | 1E+05 | 92676 | 124852 |  | 48541 | 77997 | 1.762 | 0.0626 | 1.762 |
| LMNA | 417 | 2E+05 | 201886 | 224733 | 2E+05 | 142672 | 35928 | 1.7201 | 0.1294 | 1.7201 |
| GAPDH | 107 | 4E+05 | 226471 | 778400 | 3E+05 | 330122 | 207652 | 1.7015 | 0.3235 | 1.7015 |
| MDH2 | 239 | 95488 |  | 104383 | 79293 | 53595 | 44371 | 1.6914 | 0.0605 | 1.6914 |
| FASN | 1582 | 2E+05 |  | 35927 | 90571 | 70946 |  | 1.6868 | 0.6373 | 1.6868 |
| TCP1 | 365 | 74982 | 87869 |  | 60219 |  | 37125 | 1.6729 | 0.1316 | 1.6729 |
| HADHA | 326 |  | 201534 | 190692 | 1E+05 | 105510 | 132670 | 1.6548 | 0.0057 | 1.6548 |
| YWHAZ | 9 | 3E+05 | 380113 | 340465 | 21905 | 399191 |  | 1.6522 | 0.4057 | 1.6522 |
| SUMO2 | 5 | 2E+05 | 253041 | 225944 | 2E+05 |  | 67544 | 1.5995 | 0.2434 | 1.5995 |
| NFKB2 | 72 | 41889 |  | 49129 | 32677 | 25410 |  | 1.5669 | 0.0849 | 1.5669 |
| CCT6A | 377 | 3E+05 | 327939 | 263815 | 2E+05 | 183117 |  | 1.5619 | 0.0228 | 1.5619 |
| ENO1 | 343 | 4E+05 | 345835 | 641200 | 3E+05 | 468065 | 193102 | 1.5384 | 0.2504 | 1.5384 |
| HNRNPH1 | 167;167 | 51890 | 61585 | 60563 | 53101 | 47184 | 12981 | 1.5366 | 0.1908 | 1.5366 |
| H1-3 | 17 | 2E+05 | 163474 | 97677 | 1E+05 | 68143 | 81476 | 1.5361 | 0.131 | 1.5361 |
| HADHA | 60 | 1E+05 |  | 148936 | 84206 | 88454 | 76749 | 1.534 | 0.0749 | 1.534 |
| ENO1 | 71 | 2E+05 |  | 334317 | 3E+05 | 217746 | 64159 | 1.5317 | 0.349 | 1.5317 |
| ALYREF | 86 | 29815 |  | 34778 |  | 23082 | 19591 | 1.5137 | 0.0688 | 1.5137 |
| DLD | 143 | 79510 |  | 60564 | 55484 | 31840 | 52220 | 1.5057 | 0.142 | 1.5057 |
| ATP5PO | 172 | 52006 | 35612 | 45256 | 34577 | 25413 | 28587 | 1.5001 | 0.0539 | 1.5001 |
| MAP1B | 2196 | 31041 |  | 33202 | 21273 |  | 22079 | 1.4819 | 0.012 | 1.4819 |
| PSMB5 | 91 | 27197 |  | 37832 | 33493 | 24993 | 7609.6 | 1.4758 | 0.3957 | 1.4758 |
| RBBP7 | 119 | 1E+06 | 988885 | 1E+06 | 1E+06 | 872449 | 131839 | 1.4673 | 0.3328 | 1.4673 |
| TUBA1B | 336;336;336;336;336;336 | | 287032 | 173647 | 2E+05 | 245954 | 72848 | 1.4627 | 0.4147 | 1.4627 |
| ECI2 | 359 | 47819 | 61108 | 60242 | 44774 | 45358 | 26944 | 1.445 | 0.0791 | 1.445 |
| ATP5F1A | 539 | 2E+05 | 227810 | 227609 | 2E+05 | 138040 |  | 1.4425 | 0.0168 | 1.4425 |
| RPL35 | 43 | 5E+05 |  | 668663 | 5E+05 | 386506 | 392646 | 1.4406 | 0.0597 | 1.4406 |
| H2AZ2 | 116;116 | 9E+05 | 897385 | 717873 | 6E+05 | 691127 | 438309 | 1.432 | 0.0563 | 1.432 |
| ECI2 | 92 | 5E+05 | 521736 | 545677 | 4E+05 | 393112 | 290478 | 1.4284 | 0.0229 | 1.4284 |
| ZYX | 279 | 6E+05 | 654976 | 680884 | 5E+05 | 364454 | 459730 | 1.4212 | 0.0265 | 1.4212 |
| NOP58 | 441 | 43002 | 43787 | 62947 | 49365 | 42048 | 16301 | 1.3901 | 0.3066 | 1.3901 |
| ATP5F1B | 133 | 2E+05 | 204150 | 226307 | 2E+05 | 152858 | 128961 | 1.3814 | 0.0232 | 1.3814 |
| RPS8 | 128 | 1E+06 | 1E+06 | 2E+06 | 1E+06 | 938321 | 1E+06 | 1.3674 | 0.0543 | 1.3674 |
| CLTA | 242 |  | 284145 | 293724 | 3E+05 | 232203 | 103001 | 1.3576 | 0.3891 | 1.3576 |
| ACAT1 | 174 | 2E+05 | 215771 | 248573 |  | 150423 | 170440 | 1.3491 | 0.1096 | 1.3491 |
| HSD17B4 | 579 | 2E+05 | 137568 | 163213 | 94062 | 115966 | 146212 | 1.3408 | 0.1003 | 1.3408 |
| HSDL2 | 42 | 1E+05 | 148995 | 146514 | 1E+05 | 102568 |  | 1.3359 | 0.0109 | 1.3359 |
| HSD17B4 | 725 |  | 18526 | 30863 | 18492 | 26399 | 10889 | 1.3281 | 0.4693 | 1.3281 |
| FLNA | 2024 | 25829 | 19996 | 16660 | 15488 | 16345 |  | 1.3086 | 0.2523 | 1.3086 |
| LDHA | 224 | 2E+05 | 177595 | 207141 | 2E+05 | 119564 | 163461 | 1.2894 | 0.0712 | 1.2894 |
| YARS1 | 146 | 62269 | 55056 | 53430 | 57688 | 44700 | 30128 | 1.2886 | 0.2042 | 1.2886 |
| CROT | 65 | 34849 | 30575 | 36312 | 32480 | 24330 | 22146 | 1.2885 | 0.1015 | 1.2885 |
| HSPA4 | 53 | 81690 |  | 91254 | 95671 |  | 39111 | 1.2831 | 0.5743 | 1.2831 |
| FLNC | 113 | 49111 | 64854 |  | 49120 | 39746 |  | 1.2825 | 0.3042 | 1.2825 |
| H2BC4 | 6;6;6;6;6 | 3E+06 | 4E+06 | 4E+06 | 3E+06 | 4E+06 | 1E+06 | 1.2779 | 0.4007 | 1.2779 |
| HSPE1 | 66 | 2E+05 | 146131 | 171175 | 1E+05 | 115479 |  | 1.274 | 0.0584 | 1.274 |
| MYH9 | 989 |  | 66566 | 72338 | 67223 | 59536 | 38156 | 1.2634 | 0.2946 | 1.2634 |
| PSMD1 | 310 | 2E+05 | 175334 | 275029 | 3E+05 | 162557 | 60421 | 1.2589 | 0.5751 | 1.2589 |
| AGPS | 347 | 2E+05 | 269530 | 311360 | 3E+05 | 187430 | 201995 | 1.2588 | 0.1488 | 1.2588 |
| PGK1 | 291 | 1E+05 |  | 88427 | 1E+05 |  | 28908 | 1.2497 | 0.7274 | 1.2497 |
| INTS4 | 26 | 4E+05 | 380811 | 478693 | 4E+05 | 333609 | 208714 | 1.2384 | 0.3652 | 1.2384 |
| TMSB10 | 26;26 | 4E+05 | 485614 | 493911 | 4E+05 | 398565 | 254390 | 1.2291 | 0.3004 | 1.2291 |
| SCP2 | 453 | 2E+05 | 211380 | 233322 | 2E+05 | 147462 |  | 1.2255 | 0.215 | 1.2255 |
| ATP5F1D | 136 | 58472 | 40448 | 60803 | 44323 | 42821 |  | 1.2219 | 0.3293 | 1.2219 |
| EEF1G | 212 | 2E+05 | 174936 | 198351 | 2E+05 | 123500 | 136098 | 1.2219 | 0.175 | 1.2219 |
| HNRNPD | 129 | 51370 | 54966 |  | 66391 | 33755 | 30412 | 1.2217 | 0.5628 | 1.2217 |
| BLVRA | 269 | 1E+05 | 138946 | 174798 | 1E+05 | 101276 | 120000 | 1.2183 | 0.2285 | 1.2183 |
| CCT2 | 272 | 30814 | 22889 | 23924 | 21177 | 21388 |  | 1.2158 | 0.2481 | 1.2158 |
| TPM4 | 11 | 49062 | 65638 | 76242 | 45230 | 59527 |  | 1.2151 | 0.4 | 1.2151 |
| FLNA | 700 | 6E+05 | 616123 | 673511 | 7E+05 | 586901 | 276615 | 1.2138 | 0.4267 | 1.2138 |
| PPM1G | 247 | 2E+05 | 211544 | 295703 | 2E+05 | 153792 |  | 1.2117 | 0.4708 | 1.2117 |
| TPR | 748 | 2E+06 | 3E+06 | 3E+06 | 3E+06 | 105639 | 3E+06 | 1.2075 | 0.6874 | 1.2075 |
| PICALM | 24 | 22199 | 24184 |  | 23494 | 15013 |  | 1.2045 | 0.4613 | 1.2045 |
| H2BC4 | 109;109;109;109;110;105;109;109;109;109;109;109;109;109 | 2E+05 | 264326 | 210502 | 3E+05 | 233197 | 64362 | 1.204 | 0.5995 | 1.204 |
| GLB1L2 | 502 | 3E+05 | 397012 | 266298 |  | 302664 | 251373 | 1.1908 | 0.3872 | 1.1908 |
| GAPDH | 139 | 3E+05 |  | 288364 | 3E+05 | 351550 | 126065 | 1.1901 | 0.6226 | 1.1901 |
| LMNB1 | 271 | 2E+05 |  | 145303 | 2E+05 | 98910 |  | 1.1832 | 0.4839 | 1.1832 |
| CCT2 | 154 | 2E+05 | 92946 | 107127 | 1E+05 | 94627 |  | 1.1731 | 0.5482 | 1.1731 |
| MYH9 | 74;78 | 4E+05 | 456159 | 570996 | 5E+05 | 401535 | 299835 | 1.1726 | 0.4366 | 1.1726 |
| MYH10 | 78 | 4E+05 | 456159 | 570996 | 5E+05 | 401535 | 299835 | 1.1726 | 0.4366 | 1.1726 |
| LDHB | 82 | 2E+05 | 258771 | 320143 | 3E+05 | 256774 | 81816 | 1.1673 | 0.669 | 1.1673 |
| TMSB10 | 15 | 1E+06 | 2E+06 | 2E+06 | 2E+06 | 1E+06 | 1E+06 | 1.1671 | 0.3631 | 1.1671 |
| HMGCL | 48 | 1E+06 | 1E+06 | 2E+06 | 1E+06 | 1E+06 | 1E+06 | 1.1652 | 0.2415 | 1.1652 |
| HNRNPA1 | 105;105 | 3E+05 | 350477 | 324756 | 4E+05 | 295173 | 192626 | 1.1652 | 0.4119 | 1.1652 |
| SND1 | 102 | 19466 |  | 27207 | 25203 | 22167 | 12922 | 1.1612 | 0.6019 | 1.1612 |
| VDAC1 | 96 | 26982 |  | 42428 | 24295 | 35654 |  | 1.1578 | 0.6706 | 1.1578 |
| ECI2 | 90 | 1E+05 |  | 143154 | 1E+05 | 102866 |  | 1.1575 | 0.3916 | 1.1575 |
| HSPB1 | 123 | 1E+05 | 107136 | 143124 | 1E+05 | 145723 | 16517 | 1.1548 | 0.7413 | 1.1548 |
| CREBBP | 1744 | 5E+05 | 612567 | 592579 | 3E+05 | 397721 | 699153 | 1.1542 | 0.5768 | 1.1542 |
| ALDOA | 230 | 4E+05 | 481381 | 373469 | 4E+05 | 511380 | 223110 | 1.1469 | 0.5772 | 1.1469 |
| HSD17B4 | 81 |  | 95776 | 132873 | 1E+05 | 86063 | 93165 | 1.1438 | 0.5126 | 1.1438 |
| LASP1 | 121 | 43072 | 31318 | 40480 | 36512 |  | 30550 | 1.1419 | 0.4205 | 1.1419 |
| QSER1 | 978 | 51286 | 42322 | 64855 | 56567 | 43075 | 40086 | 1.1341 | 0.4928 | 1.1341 |
| CCT4 | 288 | 62904 | 70684 | 80254 | 91163 | 67796 | 29645 | 1.1338 | 0.6748 | 1.1338 |
| ACOX1 | 267 |  | 57499 | 56100 | 54415 | 65160 | 31937 | 1.1247 | 0.6528 | 1.1247 |
| PKM | 3 |  | 21699 | 15397 | 12017 |  | 21193 | 1.117 | 0.7603 | 1.117 |
| ARFGAP1 | 231 |  | 22305 | 24718 | 31024 | 13759 | 18499 | 1.1146 | 0.7425 | 1.1146 |
| PGP | 300 | 1E+05 | 111204 | 119788 | 1E+05 | 89766 | 87559 | 1.1144 | 0.4106 | 1.1144 |
| NAA50 | 34 | 2E+06 | 2E+06 | 2E+06 | 2E+06 | 2E+06 | 2E+06 | 1.1142 | 0.1579 | 1.1142 |
| LANCL1 | 128 | 20984 | 22427 |  | 23175 |  | 15802 | 1.1138 | 0.6149 | 1.1138 |
| RPS18 | 94 | 15772 |  | 33226 | 27049 | 16969 |  | 1.1131 | 0.8279 | 1.1131 |
| LACTB2 | 102 | 74344 | 70676 |  | 72448 | 58774 |  | 1.1051 | 0.4326 | 1.1051 |
| RNF40 | 20 | 4E+05 | 363944 | 360273 | 4E+05 |  | 290413 | 1.1019 | 0.444 | 1.1019 |
| HNRNPM | 698 | 6E+05 | 652358 | 743924 | 7E+05 | 506186 | 609344 | 1.1013 | 0.435 | 1.1013 |
| FLNA | 994 | 67494 | 66897 | 71884 | 68554 | 56478 |  | 1.0998 | 0.2954 | 1.0998 |
| ENAH | 461 | 67095 | 60475 | 75631 | 81913 | 52339 | 51171 | 1.0959 | 0.6178 | 1.0959 |
| FABP5 | 72 |  | 15100 | 36142 | 32024 |  | 14883 | 1.0924 | 0.8878 | 1.0924 |
| CNN3 | 23 | 1E+05 |  | 182558 | 2E+05 | 145524 | 128634 | 1.0907 | 0.5891 | 1.0907 |
| EEA1 | 700 | 24328 |  | 32706 | 30326 | 22330 |  | 1.0831 | 0.7418 | 1.0831 |
| EED | 19 |  | 76515 | 103839 | 92428 | 74187 |  | 1.0825 | 0.7164 | 1.0825 |
| PSMA6 | 104 |  | 192702 | 178703 | 2E+05 | 179033 | 148966 | 1.0812 | 0.4453 | 1.0812 |
| LDHA | 126 | 8E+05 | 882609 | 853295 | 1E+06 | 600477 | 770134 | 1.0785 | 0.6063 | 1.0785 |
| ARCN1 | 335 |  | 26900 | 39988 | 36536 | 30327 | 26180 | 1.0783 | 0.7228 | 1.0783 |
| GOT2 | 296 |  | 42389 | 76369 | 56048 | 54696 |  | 1.0724 | 0.8356 | 1.0724 |
| BRD8 | 85 | 1E+05 | 128603 | 172527 |  | 142453 | 109052 | 1.0712 | 0.7779 | 1.0712 |
| HSPA8 | 348;351 | | 78175 | 80343 | 55556 | 93104 |  | 1.0663 | 0.8178 | 1.0663 |
| EEF1A1 | 439;439 | 2E+05 | 226177 | 281358 | 2E+05 | 181836 |  | 1.062 | 0.7976 | 1.062 |
| YEATS4 | 131 | 1E+05 | 147417 | 173334 | 2E+05 | 166791 | 72405 | 1.0609 | 0.8282 | 1.0609 |
| GSTK1 | 158 | 90082 | 103328 |  | 1E+05 | 70787 |  | 1.0601 | 0.8223 | 1.0601 |
| JPT2 | 142 | 4E+05 | 372879 |  | 4E+05 | 334394 | 346731 | 1.0566 | 0.2763 | 1.0566 |
| PTMA | 103 | 2E+05 | 214996 | 199169 | 2E+05 | 200527 | 182929 | 1.0484 | 0.4161 | 1.0484 |
| H2BC13 | 6 | 1E+05 |  | 169964 | 1E+05 | 133051 |  | 1.0479 | 0.8235 | 1.0479 |
| ANXA1 | 97 | 2E+05 | 264185 | 256719 | 3E+05 | 239744 | 184636 | 1.044 | 0.7659 | 1.044 |
| PDLIM2 | 282 | 6E+06 | 8E+06 | 1E+07 | 9E+06 | 7E+06 | 6E+06 | 1.0382 | 0.8358 | 1.0382 |
| PDLIM1 | 309 | 56216 | 56751 | 68102 | 74419 | 58427 | 41621 | 1.0378 | 0.8402 | 1.0378 |
| TALDO1 | 286 | 58821 | 47506 | 69074 | 69566 | 54715 | 44981 | 1.0363 | 0.8397 | 1.0363 |
| MED6 | 241 | 1E+05 | 120018 | 156473 | 2E+05 | 109138 | 125280 | 1.0361 | 0.8148 | 1.0361 |
| PSMD1 | 838 | 36187 |  | 45981 | 49069 | 35903 | 34079 | 1.0353 | 0.8564 | 1.0353 |
| NNMT | 39 |  | 225801 | 282913 | 3E+05 | 303935 | 152691 | 1.034 | 0.9049 | 1.034 |
| TPR | 1128 | 37948 | 35905 | 52043 | 66924 | 33904 | 21044 | 1.033 | 0.9311 | 1.033 |
| PTPN11 | 482 |  | 67796 | 69925 | 73477 | 66670 | 59874 | 1.0328 | 0.6987 | 1.0328 |
| ANXA6 | 63 | 19640 |  | 23136 | 25523 | 16212 |  | 1.0249 | 0.9262 | 1.0249 |
| H2BC5 | 6 | 4E+05 | 483539 | 444340 | 4E+05 | 467698 |  | 1.024 | 0.8633 | 1.024 |
| ALDOA | 13 | 5E+05 | 640982 | 712591 | 7E+05 | 724573 | 376679 | 1.0225 | 0.9205 | 1.0225 |
| PSMB7 | 249 | 49760 |  | 69416 | 61679 | 54917 |  | 1.0221 | 0.9126 | 1.0221 |
| EPRS1 | 300 | 88233 |  | 88726 | 84828 | 106574 | 68680 | 1.0206 | 0.9077 | 1.0206 |
| EHHADH | 280 |  | 150900 | 174959 | 2E+05 | 113492 | 191545 | 1.0137 | 0.9499 | 1.0137 |
| EHHADH | 346 | 4E+05 | 396787 | 450207 | 4E+05 | 396489 | 383906 | 1.0126 | 0.8549 | 1.0126 |
| NFKB1 | 76 | 15527 | 5774.8 | 22973 | 14622 | 14598 |  | 1.0102 | 0.983 | 1.0102 |
| SF3A1 | 531 | 44951 | 46899 | 61518 | 59737 | 41572 |  | 1.0092 | 0.964 | 1.0092 |
| XRCC6 | 461 | 8E+05 | 893228 | 906778 | 9E+05 | 797222 |  | 1.009 | 0.9105 | 1.009 |
| SGTA | 137 | 24351 | 24819 | 19948 |  | 15374 | 30357 | 1.0076 | 0.9785 | 1.0076 |
| ENO1 | 193 | 2E+05 | 218378 | 267963 | 4E+05 | 241086 | 121944 | 1.0053 | 0.9867 | 1.0053 |
| H1-2 | 21 | 12048 | 19195 | 26672 | 20943 | 17639 |  | 1.0007 | 0.9982 | 1.0007 |
| MAP7D2 | 402 |  | 144390 | 161952 | 2E+05 | 152789 | 118121 | 0.9979 | 0.9913 | 0.9979 |
| KIAA0232 | 787 |  | 14980 | 26894 | 24671 |  | 17353 | 0.9964 | 0.9924 | 0.9964 |
| EIF4A1 | 309 | 39290 | 51478 | 60157 | 49769 | 64422 | 37312 | 0.9962 | 0.9854 | 0.9962 |
| GAPDH | 84 | 3E+05 | 446207 | 392168 | 6E+05 | 474978 | 68694 | 0.9945 | 0.9905 | 0.9945 |
| KMT2D | 3079 | | 86057 | 96869 | 1E+05 | 85025 | 81216 | 0.9915 | 0.9539 | 0.9915 |
| ALDOA | 342 | 86839 |  | 122189 | 97785 | 113720 |  | 0.9883 | 0.9549 | 0.9883 |
| PRDX1 | 7 | 9E+05 | 1E+06 | 1E+06 | 1E+06 | 974363 | 903950 | 0.9847 | 0.924 | 0.9847 |
| GARS1 | 219 | 38261 |  | 47624 | 63365 |  | 23875 | 0.9845 | 0.9764 | 0.9845 |
| UBA52 | 88 | 2E+05 | 185164 |  | 2E+05 | 159944 | 191778 | 0.9845 | 0.8741 | 0.9845 |
| SPTBN1 | 2344 | 33822 | 42412 | 38364 | 58698 |  | 19527 | 0.9767 | 0.9551 | 0.9767 |
| HADHA | 406 | 1E+05 | 112556 | 135900 | 89094 |  | 149937 | 0.9755 | 0.918 | 0.9755 |
| DYNC1H1 | 4283 | 3E+05 | 297780 | 343171 | 4E+05 | 299922 | 299463 | 0.9731 | 0.7642 | 0.9731 |
| MYH9 | 355 | 77842 | 64336 | 51955 | 1E+05 | 56255 | 17589 | 0.9721 | 0.9572 | 0.9721 |
| HSD17B4 | 663 | 1E+05 | 104394 | 160358 | 1E+05 | 80271 | 194202 | 0.969 | 0.9209 | 0.969 |
| YBX1 | 170 |  | 38893 | 48305 | 59452 | 45668 | 31208 | 0.9594 | 0.8786 | 0.9594 |
| GNG5 | 11 | 15455 | 12434 | 20608 |  | 19613 | 14093 | 0.9592 | 0.8646 | 0.9592 |
| PSMA4 | 176 | 60876 | 52580 |  | 74387 | 64459 | 38607 | 0.959 | 0.8746 | 0.959 |
| ACOX1 | 437 | 1E+05 | 180817 | 212137 | 2E+05 | 202057 | 105592 | 0.9573 | 0.8746 | 0.9573 |
| DCPS | 10 | 2E+05 | 153765 | 193164 | 2E+05 | 183247 | 103020 | 0.9554 | 0.8583 | 0.9554 |
| FASN | 1704 | | 322888 | 389654 | 4E+05 | 325274 | 352856 | 0.9526 | 0.7579 | 0.9526 |
| HSPA8 | 601 | 3E+05 | 415754 | 442987 | 5E+05 | 368685 | 389711 | 0.9495 | 0.7035 | 0.9495 |
| NACA | 2005;142 | 5E+05 | 458102 | 475714 | 6E+05 | 481127 | 424867 | 0.9484 | 0.6417 | 0.9484 |
| NPM1 | 32 | 1E+05 | 135349 | 147563 | 2E+05 | 152550 | 133642 | 0.9471 | 0.4139 | 0.9471 |
| ACTB | 326;327;328;326;328;328 | 2E+06 | 2E+06 | 2E+06 | 2E+06 | 2E+06 | 1E+06 | 0.9465 | 0.7173 | 0.9465 |
| S100A11 | 3 |  | 801333 | 738338 | 1E+06 | 1E+06 | 137602 | 0.9464 | 0.9269 | 0.9464 |
| TPR | 755 | 83865 | 104717 | 87888 | 86195 | 105639 | 101027 | 0.944 | 0.5628 | 0.944 |
| SON | 2055 | 20387 |  | 36503 | 33209 | 27227 |  | 0.9413 | 0.8557 | 0.9413 |
| ALMS1 | 3058 | 14704 | 31789 | 28185 |  | 26925 | 26030 | 0.9401 | 0.8287 | 0.9401 |
| CREBBP | 1239 | 2E+05 | 103078 | 197140 | 2E+05 | 129304 |  | 0.9381 | 0.8287 | 0.9381 |
| YWHAG | 69 | 46802 | 42229 | 71163 | 77541 | 67126 | 26839 | 0.934 | 0.8433 | 0.934 |
| TPR | 713 | 2E+05 | 228363 | 299700 | 3E+05 | 231180 | 230217 | 0.9329 | 0.708 | 0.9329 |
| HSP90AA1 | 407;399;341 | 4E+05 | 390436 | 413099 | 6E+05 | 542050 | 183955 | 0.9305 | 0.8193 | 0.9305 |
| PAICS | 283 | 74859 |  | 89143 | 1E+05 | 76173 |  | 0.9296 | 0.7006 | 0.9296 |
| NCL | 467 | 78858 |  | 99052 | 97423 | 69710 | 120243 | 0.9286 | 0.7586 | 0.9286 |
| TMSB4X | 4 | 4E+05 | 326888 | 371753 | 5E+05 | 447853 | 199012 | 0.9274 | 0.7784 | 0.9274 |
| ATRX | 967 | 42521 | 55356 | 75934 | 45995 |  | 79218 | 0.9254 | 0.8084 | 0.9254 |
| FASN | 1239 | 1E+05 | 110784 | 174031 | 2E+05 | 157032 | 131998 | 0.9235 | 0.6102 | 0.9235 |
| HNRNPA2B1 | 112 | 6E+05 | 743632 | 807958 | 1E+06 | 814003 | 538779 | 0.9215 | 0.6927 | 0.9215 |
| HNRNPA3 | 199 |  | 36868 | 33977 |  | 47412 | 29707 | 0.9186 | 0.7599 | 0.9186 |
| EEF2 | 272 |  | 285429 | 294047 | 4E+05 | 272988 |  | 0.9185 | 0.6081 | 0.9185 |
| EPRS1 | 1389 | 79799 | 75576 | 95425 | 1E+05 |  | 66732 | 0.9164 | 0.7265 | 0.9164 |
| VIM | 292;288 | 73193 | 82690 | 102749 | 1E+05 |  | 72930 | 0.9092 | 0.6934 | 0.9092 |
| PTGES3 | 33 | 4E+05 | 390625 | 469373 | 5E+05 |  | 394578 | 0.9088 | 0.5345 | 0.9088 |
| AASDHPPT | 151 | 4E+05 | 402962 | 481955 | 5E+05 | 399638 |  | 0.9079 | 0.5461 | 0.9079 |
| NAE1 | 378 | 24847 | 41137 | 41504 | 46617 | 32409 |  | 0.9068 | 0.7052 | 0.9068 |
| MYH9 | 555 | 1E+05 | 139355 |  | 2E+05 | 181846 | 124280 | 0.9065 | 0.5604 | 0.9065 |
| CFL1 | 144 | 1E+05 | 162702 | 230365 | 2E+05 | 200884 | 131820 | 0.9062 | 0.7102 | 0.9062 |
| H2BC4 | 47;47;47;47;48;47;47;47;47;47;47;47;47 | 10979 | 8136.6 | 13026 | 15717 |  | 7957 | 0.9051 | 0.7644 | 0.9051 |
| INF2 | 666 |  | 43083 | 49051 | 58901 | 43010 |  | 0.9041 | 0.6228 | 0.9041 |
| HMGN2 | 82 |  | 38074 | 40309 | 54147 | 58904 | 18621 | 0.8929 | 0.7935 | 0.8929 |
| ANXA1 | 312 | 3E+05 | 367926 | 429729 | 5E+05 | 493512 | 249605 | 0.8883 | 0.6352 | 0.8883 |
| MYH9 | 545 | 15011 | 13787 | 16652 | 19902 | 16466 | 14850 | 0.8874 | 0.3226 | 0.8874 |
| ANXA2 | 47;47 | | 93315 | 144324 | 1E+05 | 109923 | 154856 | 0.886 | 0.5901 | 0.886 |
| YWHAZ | 157 | 2E+05 | 176669 |  | 2E+05 | 179580 |  | 0.8848 | 0.3211 | 0.8848 |
| RBM14 | 164 | 1E+05 | 140932 | 138654 |  | 150702 | 140563 | 0.8832 | 0.3374 | 0.8832 |
| CLIC1 | 131 | 2E+05 | 272155 | 289505 | 4E+05 | 253108 | 274121 | 0.8804 | 0.4385 | 0.8804 |
| FLNA | 2569 | 1E+05 | 171622 |  | 2E+05 | 199543 | 102636 | 0.8802 | 0.7133 | 0.8802 |
| PHYH | 120 | 38970 |  | 44675 | 40439 | 52577 | 49588 | 0.8798 | 0.3499 | 0.8798 |
| HBD | 145;145 | 1E+05 | 115042 | 171326 | 2E+05 | 140187 | 135949 | 0.8775 | 0.5497 | 0.8775 |
| ZFR | 509 | 2E+05 |  | 481087 | 4E+05 | 288119 | 522264 | 0.872 | 0.704 | 0.872 |
| EP300 | 1760;1797 | 5E+05 |  | 656653 | 9E+05 | 713461 | 435376 | 0.8705 | 0.6616 | 0.8705 |
| PKM | 433 | 49413 | 77892 | 79368 | 1E+05 | 83412 | 27537 | 0.8701 | 0.7511 | 0.8701 |
| HSPA8 | 187;188 | 30898 |  | 36244 | 45435 | 31964 |  | 0.8675 | 0.5525 | 0.8675 |
| RUVBL2 | 365 | 48415 | 42083 | 56877 | 69895 |  | 44058 | 0.8622 | 0.5305 | 0.8622 |
| RNPS1 | 218 | 55796 | 54784 | 71728 | 86353 |  | 54653 | 0.8619 | 0.5307 | 0.8619 |
| MYH9 | 1828 | | 37011 | 33406 | 39982 | 45084 | 38130 | 0.8574 | 0.1461 | 0.8574 |
| MAP3K7 | 474 | 3E+05 | 301576 |  | 3E+05 | 156426 | 484151 | 0.8541 | 0.726 | 0.8541 |
| VIM | 104;109 | 3E+05 | 318535 | 384222 | 5E+05 | 385652 | 314855 | 0.8541 | 0.3257 | 0.8541 |
| HSP90AA1 | 283;282;275;197 | 1E+05 | 165920 | 226761 |  | 245348 | 176428 | 0.851 | 0.4953 | 0.851 |
| ITGB1 | 794 |  | 55717 | 83163 | 89724 | 73536 |  | 0.8507 | 0.5242 | 0.8507 |
| EP300 | 1203 | 2E+05 | 120333 | 220131 | 2E+05 | 239208 |  | 0.8497 | 0.5866 | 0.8497 |
| KHDRBS3 | 152 | 15161 |  | 36839 | 46701 |  | 14548 | 0.849 | 0.8337 | 0.849 |
| LDHB | 244 | 23763 |  | 38678 | 45602 | 27650 | 37771 | 0.8436 | 0.554 | 0.8436 |
| EIF4A1 | 146 | 1E+05 | 121044 | 121634 | 2E+05 | 137583 | 111297 | 0.8425 | 0.2763 | 0.8425 |
| BRD8 | 481 | 1E+05 | 111553 | 150671 | 2E+05 |  | 108286 | 0.8422 | 0.5728 | 0.8422 |
| DARS1 | 126 | 9203 |  | 20700 | 19682 | 15860 |  | 0.8414 | 0.6874 | 0.8414 |
| MYH9 | 1014 | 89327 | 96601 | 120726 | 2E+05 | 105331 | 103039 | 0.8412 | 0.3843 | 0.8412 |
| TMSB4X | 12 | 5E+05 | 488092 | 495052 | 6E+05 | 523060 | 628829 | 0.8404 | 0.0414 | 0.8404 |
| UQCRH | 42;42 | 27369 |  | 35671 | 47405 | 27613 |  | 0.8403 | 0.6329 | 0.8403 |
| ENO1 | 335 | 1E+06 | 2E+06 | 2E+06 | 2E+06 | 2E+06 | 2E+06 | 0.8396 | 0.2289 | 0.8396 |
| VIM | 439 | 68258 |  | 60720 | 1E+05 | 73098 | 36008 | 0.8363 | 0.7225 | 0.8363 |
| MED6 | 236 | 3E+05 | 347004 | 424771 | 6E+05 | 460378 | 209991 | 0.835 | 0.6032 | 0.835 |
| ANXA5 | 97 | 34379 | 31065 |  | 46784 | 32008 |  | 0.8306 | 0.4711 | 0.8306 |
| STIP1 | 284 |  | 30514 | 39025 | 51224 |  | 32608 | 0.8295 | 0.5573 | 0.8295 |
| STMN1 | 119 | 2E+05 | 208375 | 275716 | 3E+05 | 305167 | 189447 | 0.8284 | 0.3935 | 0.8284 |
| DOCK7 | 1962 | 1E+05 | 138070 | 185726 | 2E+05 |  | 137761 | 0.8265 | 0.4933 | 0.8265 |
| SMNDC1 | 219 | 1E+05 | 131473 | 157603 | 2E+05 | 125230 | 191470 | 0.8263 | 0.2924 | 0.8263 |
| PFN1 | 105 | 2E+05 | 228903 | 321687 | 4E+05 | 361335 | 128485 | 0.8249 | 0.6103 | 0.8249 |
| AHNAK | 2561 | 41944 | 74310 | 87553 |  | 69662 | 95348 | 0.8234 | 0.5178 | 0.8234 |
| SQOR | 135 | 44708 | 70057 | 14690 | 62118 | 42871 |  | 0.822 | 0.698 | 0.822 |
| MYH9 | 1370 | | 28018 | 34664 | 51583 | 40194 | 23180 | 0.8179 | 0.5689 | 0.8179 |
| NEDD8 | 54 | 32912 | 40489 | 43306 | 51055 | 48243 | 43484 | 0.8174 | 0.0847 | 0.8174 |
| VIM | 294;290 | 7E+05 | 792065 | 861822 | 1E+06 | 814162 | 927731 | 0.8156 | 0.1779 | 0.8156 |
| TCEA1 | 265 |  | 21175 | 30095 | 35916 | 27264 |  | 0.8115 | 0.4389 | 0.8115 |
| PTMA | 15 | 5E+06 | 6E+06 | 7E+06 | 9E+06 | 7E+06 | 7E+06 | 0.8109 | 0.1542 | 0.8109 |
| DBI | 55 | 2E+06 | 2E+06 | 2E+06 | 1E+06 | 2E+06 | 3E+06 | 0.8078 | 0.4987 | 0.8078 |
| PGAM1 | 100;100;100 | 58577 |  | 86561 | 88656 | 91515 |  | 0.8056 | 0.3391 | 0.8056 |
| NPM1 | 257 | 1E+05 |  | 166674 | 2E+05 | 167546 |  | 0.8046 | 0.3143 | 0.8046 |
| CBR1 | 148 |  | 24131 | 36119 | 38913 | 35967 |  | 0.8046 | 0.3577 | 0.8046 |
| PTMS | 4 | 1E+05 |  | 161074 | 2E+05 | 126621 |  | 0.804 | 0.5834 | 0.804 |
| VCP | 109 | 62391 | 84392 | 98335 | 96780 | 71618 | 136668 | 0.8035 | 0.408 | 0.8035 |
| MVP | 745 | 70085 | 57002 |  | 1E+05 | 76193 | 59124 | 0.8026 | 0.423 | 0.8026 |
| RAI14 | 936 | 58843 | 62711 | 70822 | 95105 | 65466 |  | 0.7987 | 0.2691 | 0.7987 |
| FLNA | 578 | 3E+05 | 383494 | 396358 | 6E+05 | 436728 | 399119 | 0.7977 | 0.1538 | 0.7977 |
| TUBAL3 | 401;394;394;394;394;394;394 | | 31971 | 41253 | 53622 | 38848 | 45955 | 0.7935 | 0.2391 | 0.7935 |
| RPS20 | 8 | 7E+05 | 753609 | 948613 | 1E+06 | 989723 | 744701 | 0.7856 | 0.2882 | 0.7856 |
| RBBP4 | 4 | 1E+05 | 400229 | 370551 | 4E+05 | 345182 |  | 0.7823 | 0.5465 | 0.7823 |
| PPIA | 82 | 4E+05 | 518804 | 524641 | 8E+05 | 601242 | 462393 | 0.7811 | 0.2877 | 0.7811 |
| ALDOA | 147;147 | 4E+05 | 385564 | 451055 |  | 508897 | 539660 | 0.7806 | 0.0297 | 0.7806 |
| VDAC1 | 224 | 1E+06 | 1E+06 | 2E+06 | 2E+06 | 1E+06 | 2E+06 | 0.7749 | 0.1972 | 0.7749 |
| VDAC2 | 74 | 4E+05 | 356501 |  | 6E+05 | 403224 | 575703 | 0.7703 | 0.2427 | 0.7703 |
| ZC3H4 | 1067 | | 10968 | 12831 | 19916 | 12755 | 13703 | 0.7698 | 0.3184 | 0.7698 |
| ZYX | 24 | 3E+05 | 307141 | 379133 | 5E+05 | 314972 |  | 0.7691 | 0.3524 | 0.7691 |
| NPM1 | 27 | 52797 | 65800 | 76950 | 90888 | 78861 |  | 0.768 | 0.1453 | 0.768 |
| VIM | 129 | 83194 |  | 73937 | 1E+05 | 86701 |  | 0.7664 | 0.2833 | 0.7664 |
| SOD2 | 68 | 2E+05 | 74881 |  | 2E+05 | 203180 | 76849 | 0.7618 | 0.576 | 0.7618 |
| PHB2 | 200 | 10080 | 14382 | 15928 | 22005 | 13497 |  | 0.7584 | 0.3511 | 0.7584 |
| GRIPAP1 | 230 | 9E+05 | 1E+06 | 1E+06 | 2E+06 | 1E+06 | 1E+06 | 0.758 | 0.035 | 0.758 |
| NCL | 116 | 2E+05 | 28294 | 29336 | 2E+05 | 159377 | 17678 | 0.7569 | 0.7284 | 0.7569 |
| MYH9 | 1492 | | 51651 | 58533 | 96695 | 81409 | 40310 | 0.7567 | 0.4776 | 0.7567 |
| ECI2 | 51 | 2E+05 | 140464 | 198059 | 2E+05 | 231021 | 259854 | 0.7558 | 0.1374 | 0.7558 |
| C14orf28 | 2 | 3E+05 | 313179 | 479481 | 5E+05 | 392575 |  | 0.7556 | 0.3342 | 0.7556 |
| PSAT1 | 127 | 70423 | 82183 |  |  | 104884 | 97500 | 0.754 | 0.0698 | 0.754 |
| PLIN3 | 65 | 2E+05 |  | 256315 | 4E+05 | 306449 | 277508 | 0.7532 | 0.1727 | 0.7532 |
| UBN2 | 1148 | 2E+05 | 240457 | 289535 | 4E+05 | 323883 | 276020 | 0.7476 | 0.1165 | 0.7476 |
| MRPL47 | 144 | 70709 | 33516 | 80981 | 69046 |  | 97154 | 0.7429 | 0.3909 | 0.7429 |
| LDHA | 243 | 67511 | 66296 | 87609 | 1E+05 | 103423 | 85025 | 0.7413 | 0.0653 | 0.7413 |
| MEAF6 | 69 | 3E+05 | 384523 | 330985 | 5E+05 | 435844 | 419353 | 0.7404 | 0.0344 | 0.7404 |
| PRDX1 | 27 | 2E+05 | 165054 | 222856 | 3E+05 | 193541 | 236159 | 0.7403 | 0.2141 | 0.7403 |
| SRSF11 | 459 |  | 27029 | 45137 | 52449 | 46234 |  | 0.7313 | 0.3003 | 0.7313 |
| CCT4 | 319 | 18180 | 5451.6 | 31657 | 48534 | 17683 | 9683 | 0.7284 | 0.6505 | 0.7284 |
| HNRNPR | 126 | 2E+05 | 220903 | 241074 | 3E+05 | 247961 |  | 0.727 | 0.1558 | 0.727 |
| PSMD12 | 221 | 14218 |  | 16372 | 23366 | 18616 | 21271 | 0.7254 | 0.059 | 0.7254 |
| NASP | 450 | 9810 |  | 12610 | 19267 | 11806 |  | 0.7215 | 0.3911 | 0.7215 |
| HSP90AA1 | 478 | 1E+05 | 112592 | 157542 | 2E+05 | 155392 |  | 0.7202 | 0.1495 | 0.7202 |
| EP300 | 1707 | 87208 | 125814 | 139613 | 2E+05 | 167844 | 130540 | 0.7197 | 0.1256 | 0.7197 |
| TLN1 | 2130;2131 | | 30258 | 35853 | 58218 | 33747 |  | 0.7189 | 0.4113 | 0.7189 |
| IRF2BP2 | 236 | 10552 |  | 26614 | 37876 | 14144 |  | 0.7145 | 0.6559 | 0.7145 |
| LRRC59 | 135 | 52686 | 42131 | 54626 | 86145 | 60887 | 64743 | 0.7057 | 0.0767 | 0.7057 |
| KDM6A | 799 |  | 57522 | 72527 | 74526 | 98566 | 103778 | 0.7046 | 0.125 | 0.7046 |
| DDX24 | 17 | 2E+05 | 171715 | 181781 | 3E+05 | 220340 | 223277 | 0.6961 | 0.0679 | 0.6961 |
| AKR1B1 | 195 |  | 42539 | 51020 | 42288 |  | 92435 | 0.6945 | 0.5033 | 0.6945 |
| MYH9 | 1793 | 91312 |  | 129463 |  | 162537 | 159383 | 0.6858 | 0.1183 | 0.6858 |
| ILK | 426 | 2E+05 |  | 299135 | 4E+05 | 450068 | 340207 | 0.6775 | 0.0679 | 0.6775 |
| ENO1 | 420 |  | 112744 | 165383 | 2E+05 | 211467 |  | 0.6743 | 0.1294 | 0.6743 |
| HNRNPK | 179 | 55524 | 64399 | 59950 | 97960 | 77780 | 91800 | 0.6723 | 0.0108 | 0.6723 |
| ASCC3 | 572 | 53315 | 62074 | 95232 | 2E+05 | 125886 | 34627 | 0.669 | 0.4157 | 0.669 |
| TPI1 | 142 | 52448 | 52374 | 55258 | 73410 | 71013 | 95002 | 0.6686 | 0.0263 | 0.6686 |
| NUDT21 | 23 | 2E+05 | 258324 | 296207 | 2E+05 | 176082 | 742683 | 0.6679 | 0.5396 | 0.6679 |
| PTMS | 92 | 15911 |  | 25341 | 30057 | 30418 | 32279 | 0.6671 | 0.0657 | 0.6671 |
| XRCC5 | 565 | 36981 | 40389 | 45767 | 54755 | 48301 | 81566 | 0.667 | 0.1227 | 0.667 |
| MYH9 | 1802 | 2E+05 | 260690 | 232121 | 4E+05 | 349334 |  | 0.6627 | 0.0057 | 0.6627 |
| RPS20 | 4 | 1E+05 | 171446 | 210008 | 3E+05 | 245746 |  | 0.6609 | 0.0596 | 0.6609 |
| HSDL2 | 49 | 13281 | 150093 | 181821 | 2E+05 | 183276 | 121561 | 0.6601 | 0.3715 | 0.6601 |
| GOT2 | 122 |  | 15503 | 21793 | 37353 | 19193 |  | 0.6596 | 0.4221 | 0.6596 |
| VIM | 235 | 2E+05 | 310655 | 365231 | 6E+05 | 449492 | 375740 | 0.6552 | 0.0767 | 0.6552 |
| HNRNPA3 | 126 | 1E+05 | 87006 | 146393 | 2E+05 | 192607 | 124785 | 0.6481 | 0.1314 | 0.6481 |
| FSCN1 | 74 | 34259 | 39112 | 58699 | 70196 | 65844 | 68048 | 0.6471 | 0.0339 | 0.6471 |
| NCOA2 | 780 | 61847 | 132232 | 146221 | 2E+05 | 191186 | 126467 | 0.6446 | 0.1609 | 0.6446 |
| MIF | 78 | 1E+05 | 120484 | 135738 | 2E+05 | 204014 | 112680 | 0.6404 | 0.1667 | 0.6404 |
| ANXA2 | 302;302 | 2E+05 |  | 251571 | 4E+05 | 320019 |  | 0.6335 | 0.1135 | 0.6335 |
| S100A6 | 47 | 1E+05 | 113673 | 151448 | 2E+05 | 172356 | 196737 | 0.6231 | 0.0222 | 0.6231 |
| CALM3 | 22;22;22 | 54839 | 51812 | 67902 | 94450 | 86920 | 101289 | 0.6175 | 0.005 | 0.6175 |
| HSP90AA1 | 112;112;107 | 45709 | 46079 |  | 84942 | 64081 |  | 0.6159 | 0.1112 | 0.6159 |
| PRKDC | 1051 | 15403 | 20891 | 29429 | 43805 |  | 27579 | 0.6138 | 0.1842 | 0.6138 |
| COPA | 271 | 21225 | 35405 | 36905 | 62173 | 46730 | 45366 | 0.6063 | 0.0511 | 0.6063 |
| CALM3 | 95;95;95 | | 104706 | 99149 | 2E+05 | 177712 | 161539 | 0.6037 | 0.0019 | 0.6037 |
| SYNRG | 744 | 51449 | 32841 | 67673 | 91657 | 76197 | 84917 | 0.6012 | 0.038 | 0.6012 |
| NSFL1C | 172 | 25338 |  | 25617 | 51006 |  | 35258 | 0.5907 | 0.1542 | 0.5907 |
| GMNN | 27 | 23661 |  | 34014 | 62284 | 31601 | 52897 | 0.5894 | 0.2019 | 0.5894 |
| RPS27A | 27;27;27;27 | 43315 | 30773 | 45114 | 96086 | 66026 | 40224 | 0.5891 | 0.1736 | 0.5891 |
| LGALS1 | 13 | 39568 | 45641 | 54102 | 1E+05 | 74324 | 53733 | 0.5809 | 0.1281 | 0.5809 |
| RAPH1 | 250 | 67272 | 69403 | 111495 | 1E+05 | 98258 | 206599 | 0.575 | 0.1599 | 0.575 |
| PCNP | 64 |  | 25234 | 38454 | 61023 | 72310 | 34251 | 0.57 | 0.2163 | 0.57 |
| RBM26 | 709 | 22459 | 25138 |  | 49521 | 37876 | 39137 | 0.5642 | 0.0325 | 0.5642 |
| UBA6 | 544 | 49512 | 72879 | 74847 | 1E+05 | 110281 |  | 0.5507 | 0.0233 | 0.5507 |
| PFN1 | 108 | 41585 | 46315 | 46507 | 89606 | 78173 | 76365 | 0.5505 | 0.0012 | 0.5505 |
| FOSL2 | 222 | 41140 | 46372 |  | 83295 | 77640 |  | 0.5438 | 0.0108 | 0.5438 |
| VIM | 334 | 24690 | 39903 | 54830 | 78539 | 86435 | 57253 | 0.5374 | 0.0497 | 0.5374 |
| C12orf57 | 101 | 13081 |  | 30833 | 58590 |  | 23649 | 0.534 | 0.4313 | 0.534 |
| EP300 | 1674;1711 | 2E+05 | 432576 | 660299 | 1E+06 | 917856 | 467237 | 0.527 | 0.1598 | 0.527 |
| VIM | 373 | 94977 | 94419 | 115581 | 2E+05 | 212371 | 159196 | 0.522 | 0.0082 | 0.522 |
| YWHAZ | 68 | 4057 | 15281 | 9470.8 | 20520 | 16341 |  | 0.521 | 0.1424 | 0.521 |
| GPI | 252 | 14181 | 16620 | 15329 | 34078 | 25016 |  | 0.5204 | 0.0271 | 0.5204 |
| SCP2 | 534 | 30939 | 15557 | 22273 | 16580 |  | 73604 | 0.5084 | 0.3881 | 0.5084 |
| PTMS | 15 | 9E+05 | 1E+06 | 2E+06 | 3E+06 | 3E+06 | 2E+06 | 0.5018 | 0.0184 | 0.5018 |
| EEF2 | 42 | 1E+05 | 90793 | 146070 | 3E+05 | 285536 | 140572 | 0.5017 | 0.0777 | 0.5017 |
| ALDOA | 42 | 4E+05 | 455756 | 689339 | 1E+06 | 1E+06 | 681754 | 0.4947 | 0.076 | 0.4947 |
| ENO1 | 92 | 43829 | 45726 |  | 99179 | 83818 |  | 0.4894 | 0.0264 | 0.4894 |
| MTHFD1 | 553 | 8201 |  | 11149 | 21394 |  | 18309 | 0.4874 | 0.0413 | 0.4874 |
| PKM | 89 |  | 29100 | 48159 | 2E+05 | 44142 | 38030 | 0.4867 | 0.4747 | 0.4867 |
| HSPA8 | 71 |  | 26380 | 30646 |  | 62444 | 56341 | 0.4801 | 0.0142 | 0.4801 |
| EP300 | 1542 | 75325 | 103929 | 99628 | 2E+05 | 196065 | 198620 | 0.4777 | 0.0004 | 0.4777 |
| EP300 | 1546 | 75325 | 103929 | 99628 | 2E+05 | 196065 | 198620 | 0.4777 | 0.0004 | 0.4777 |
| ACTB | 50;51;52;50;52;52 | | 9877.6 | 10392 | 12868 | 21129 | 29993 | 0.4751 | 0.1779 | 0.4751 |
| TPI1 | 194 | 69897 | 67622 | 93098 | 2E+05 | 153444 | 138471 | 0.472 | 0.0111 | 0.472 |
| NT5DC1 | 171 | 20987 | 27420 |  | 39465 | 25948 | 100915 | 0.4366 | 0.3726 | 0.4366 |
| PGK1 | 30 | 1E+05 | 126351 | 153143 | 4E+05 | 355285 | 155136 | 0.4285 | 0.0967 | 0.4285 |
| PRKDC | 309 | 21636 | 25731 | 35473 | 77324 | 53843 |  | 0.421 | 0.034 | 0.421 |
| TMSB4X | 26 |  | 75943 | 88183 | 67317 | 61336 | 456361 | 0.4208 | 0.5513 | 0.4208 |
| ING4 | 129 | 1E+05 | 141317 | 137494 | 3E+05 | 318151 | 319346 | 0.4141 | 2E-05 | 0.4141 |
| TKT | 204 | 24658 | 26691 | 34785 | 81885 | 65776 | 62732 | 0.4094 | 0.0035 | 0.4094 |
| ING4 | 127 | 1E+05 | 141317 | 137494 | 3E+05 | 318151 | 319346 | 0.4068 | 3E-05 | 0.4068 |
| ALDOA | 153 | 22375 |  | 41168 | 1E+05 | 76909 | 49534 | 0.3876 | 0.1619 | 0.3876 |
| MYH9 | 682;689 | 49962 | 47410 | 53691 | 2E+05 |  | 92635 | 0.3875 | 0.065 | 0.3875 |
| SF3A1 | 486 |  | 11769 | 95563 | 2E+05 | 134622 |  | 0.3752 | 0.1716 | 0.3752 |
| ID1 | 20 | 54015 | 53238 | 20405 | 1E+05 | 106802 | 105106 | 0.3748 | 0.0061 | 0.3748 |
| FABP5 | 61 | 17087 | 22728 | 27254 | 31865 | 26108 | 127382 | 0.3618 | 0.2978 | 0.3618 |
| NONO | 336 | 2071 |  | 9194 |  | 8665.3 | 25413 | 0.3306 | 0.3367 | 0.3306 |
| TMSB4X | 39 | 19616 | 16957 |  | 50952 |  | 65966 | 0.3128 | 0.0342 | 0.3128 |
| PSMC6 | 206 | 12059 | 29538 | 5618.8 | 85812 | 38075 | 35369 | 0.2965 | 0.1049 | 0.2965 |
| HNRNPK | 163 | 45814 | 50950 | 36838 | 98330 | 156515 | 198313 | 0.2948 | 0.022 | 0.2948 |
| YAP1 | 280 |  | 127576 | 188138 | 7E+05 | 716589 | 339101 | 0.2668 | 0.0779 | 0.2668 |
| PCNA | 80 | 2E+05 | 167114 | 257657 | 8E+05 | 736459 | 697885 | 0.2585 | 0.0003 | 0.2585 |
| CYB5R3 | 42 | 21201 | 34088 |  | 2E+05 | 127396 | 53365 | 0.2418 | 0.1292 | 0.2418 |
| CCT6A | 365 | 33569 |  | 53436 | 2E+05 | 177364 | 197129 | 0.2163 | 0.0047 | 0.2163 |
| MITF | 45 | 20970 | 19695 |  | 1E+05 |  | 75453 | 0.2134 | 0.0635 | 0.2134 |
| HNRNPA2B1 | 59 |  | 149497 | 228820 | 1E+06 | 931608 |  | 0.1941 | 0.0055 | 0.1941 |
| THUMPD1 | 15 |  | 8504.6 | 16555 | 77698 | 83763 | 74807 | 0.1591 | 0.0007 | 0.1591 |
| CFL1 | 92;92 | 11170 | 7957.9 | 26833 |  | 94574 | 114999 | 0.1462 | 0.0036 | 0.1462 |
| TCP1 | 400 | 91695 |  | 251399 | 2E+06 | 1E+06 | 1E+06 | 0.1232 | 0.0122 | 0.1232 |
| MYC | 148 | 33890 |  | 61056 | 3E+05 | 264116 | 721503 | 0.108 | 0.1232 | 0.108 |
| VDAC3 | 12 | 45251 | 48639 |  | 5E+05 | 464311 | 458765 | 0.0968 | 0.0007 | 0.0968 |
| HNRNPA1 | 350 | 28357 | 31123 | 72991 | 9E+05 | 687323 | 859293 | 0.0546 | 0.0003 | 0.0546 |
| GAPDH | 194 | 1E+05 |  | 222129 | 4E+06 | 3E+06 | 3E+06 | 0.0486 | 0.0015 | 0.0486 |
